# *HNF1B* variants associate with promoter methylation and regulate gene networks activated in prostate and ovarian cancer

**DOI:** 10.18632/oncotarget.12543

**Published:** 2016-10-09

**Authors:** Helen Ross-Adams, Stephen Ball, Kate Lawrenson, Silvia Halim, Roslin Russell, Claire Wells, Siri H. Strand, Torben F. Ørntoft, Melissa Larson, Sebastian Armasu, Charles E. Massie, Mohammad Asim, Martin M. Mortensen, Michael Borre, Kathryn Woodfine, Anne Y. Warren, Alastair D. Lamb, Jonathan Kay, Hayley Whitaker, Antonio Ramos-Montoya, Adele Murrell, Karina D. Sørensen, Brooke L. Fridley, Ellen L. Goode, Simon A. Gayther, John Masters, David E. Neal, Ian G. Mills

**Affiliations:** ^1^ Cancer Research UK Cambridge Institute, University of Cambridge, Cambridge, UK; ^2^ Prostate Cancer Research Centre, University College London, London, UK; ^3^ Department of Preventive Medicine, University of Southern California Keck School of Medicine, Los Angeles, CA, USA; ^4^ Division of Cancer Studies, King's College London, London, UK; ^5^ Department of Molecular Medicine, Aarhus University Hospital, Denmark; ^6^ Mayo Clinic, SW, Rochester, MN, USA; ^7^ Department of Biology and Biochemistry, University of Bath, Centre for Regenerative Medicine, Claverton Down, Bath, UK; ^8^ Department of Urology, Aarhus University Hospital, Aarhus, Denmark; ^9^ Department of Pathology, Addenbrooke's Hospital, Cambridge, UK; ^10^ Department of Urology, Addenbrooke's Hospital, Cambridge, UK; ^11^ Department of Biostatistics, University of Kansas Medical Center, Kansas City, KS, USA; ^12^ Prostate Cancer Research Group, Centre for Molecular Medicine Norway, Nordic EMBL Partnership, University of Oslo and Oslo University Hospital, Oslo, Norway; ^13^ Departments of Cancer Prevention and Urology, Institute of Cancer Research and Department of Urology, Oslo University Hospital, Oslo, Norway; ^14^ Prostate Cancer UK/Movember Centre of Excellence for Prostate Cancer Research, Centre for Cancer Research and Cell Biology, Queens University Belfast, Belfast, UK; ^15^ Molecular Diagnostics and Therapeutics Group, University College London, London, UK

**Keywords:** HNF1B, eQTL, prostate, ovarian, cancer

## Abstract

Two independent regions within *HNF1B* are consistently identified in prostate and ovarian cancer genome-wide association studies (GWAS); their functional roles are unclear. We link prostate cancer (PC) risk SNPs rs11649743 and rs3760511 with elevated *HNF1B* gene expression and allele-specific epigenetic silencing, and outline a mechanism by which common risk variants could effect functional changes that increase disease risk: functional assays suggest that *HNF1B* is a pro-differentiation factor that suppresses epithelial-to-mesenchymal transition (EMT) in unmethylated, healthy tissues. This tumor-suppressor activity is lost when *HNF1B* is silenced by promoter methylation in the progression to PC. Epigenetic inactivation of *HNF1B* in ovarian cancer also associates with known risk SNPs, with a similar impact on EMT. This represents one of the first comprehensive studies into the pleiotropic role of a GWAS-associated transcription factor across distinct cancer types, and is the first to describe a conserved role for a multi-cancer genetic risk factor.

## INTRODUCTION

**Figure 1:**
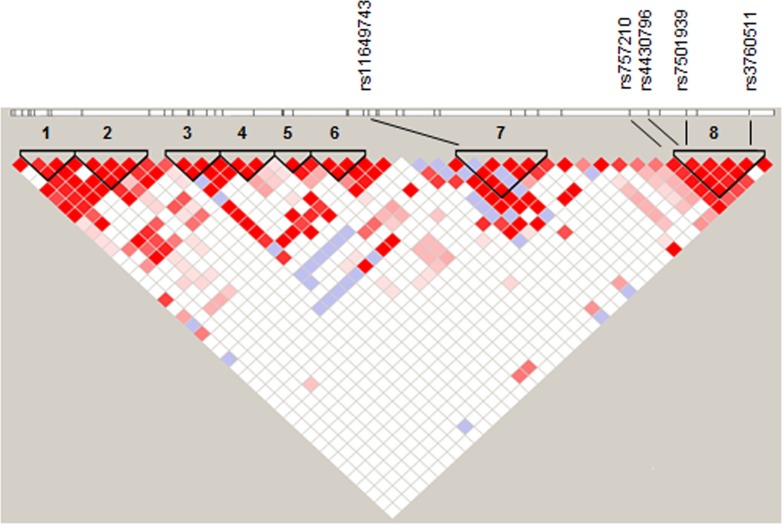
Overall LD within *HNF1B* highlighting eight regions (black triangles), with rs11649743 in region 7 and rs4430796, rs7501939 and rs3760511 in region 8 Ovarian cancer risk SNP rs757210 lies immediately adjacent to region 8. D′ values were derived from 1000 genomes project CEU data (http://www.1000genomes.org/), and linkage plots generated in Haploview using 95 % confidence intervals (Gabriel 2002).

Genome-wide association studies (GWAS) and fine-mapping have identified several distinct variants within the hepatocyte nuclear factor 1b (*HNF1B*) gene associated with increased risk of prostate cancer [1–8] and high-grade serous and clear cell epithelial ovarian cancer (OC) [9, 10]. It has also been associated with both type I and II endometrial cancer risk in a large meta-analysis [11]. *HNF1B* has three main regions of risk association (Figure 1) in which the four most significant risk variants for these two cancer types lie: rs11649743 in linkage block 7, rs4430796 and rs7501939 (all prostate) are in strong linkage disequilibrium in region 8 (r^2^ = 0.764) (Table 1), with the most significant SNP for serous invasive ovarian cancer risk rs757210 immediately adjacent. Prostate cancer (PC) risk SNP rs3760511 is within 1kb of the transcription start site (TSS) and was identified in a GWAS of four European populations; although not the most statistically significant marker, the rs-3760511-C allele is associated with an odds ratio of 1.16 [2].

**Table 1:** Pair-wise estimates of LD between each of the five markers studied (r2; CEU population), confirming three distinct groupings of the SNPs studied

	rs11649743	rs757210	rs4430796	rs7501939	rs3760511
**rs11649743**	1	0.005	0.014	0.025	0.116
**rs757210**		1	0.543	0.739	0.163
**rs4430796**			1	0.764	0.337
**rs7501939**				1	0.257
**rs3760511**					1

*HNF1B* encodes a transcription factor with a central role in vertebrate development and embryonic survival [12]. Rare exonic mutations result in uro-genital defects, pancreatic atrophy and maturity onset diabetes of the young 5 (MODY5) [13], but its role in tumor development is yet to be described. Based on the identification of several cancer-associated SNPs within *HNF1B*, we aimed to define the function of *HNF1B* in this context. *In vitro* functional assays together with gene expression data from matched cancer and non-cancer patient tissue suggest that *HNF1B* exerts a tumor-suppressive effect when over-expressed in cancer cells. This protective effect is lost following DNA silencing by promoter methylation in the development of prostate cancer. To determine whether *HNF1B* expression can act as a brake on cancer development, we assessed the phenotypic effects of ectopic *HNF1B* over-expression in cancer cells, and observed a reversal of epithelial-to-mesenchymal transition (EMT). This represents one of the first comprehensive studies to demonstrate a functional role for a GWAS-identified target from prostate cancer, and to show that a similar mechanism operates in other cancer types. Our data support an emerging hypothesis that common functional mechanisms underlie the biology of neoplastic development at pleotropic risk loci identified by GWAS [14]. This work highlights the potential relevance of GWAS-findings across different but related complex disease types, providing a starting point for other functional follow-up studies and further collaborations.

## RESULTS

### *HNF1B* expression correlates with risk SNP genotype in prostate tissues

**Figure 2:**
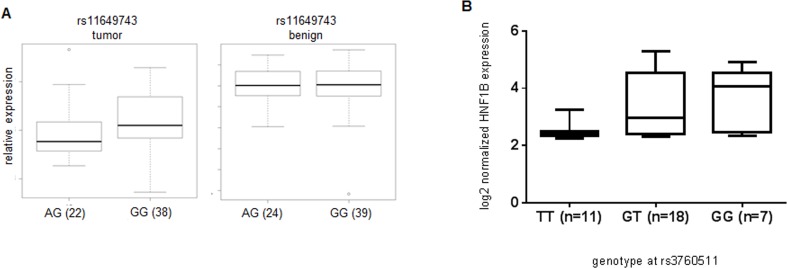
Association between genotype and increased gene expression at rs11649743 with *HNF1B* levels in prostate tumor tissue, but not benign (Kruskal-Wallis *p* = 0.038) in British samples (A), between rs3760511-G and *HNF1B* levels in laser-capture micro-dissected Danish prostate tumor samples (Kruskal-Wallis *p* = 0.018) (B)

To determine whether any of the known, significant PC risk SNPs correlated with *HNF1B* expression levels, we genotyped each SNP in 65 British patients, and compared these to mRNA levels of *HNF1B* in tumor tissue and, where possible, matched non-tumor tissue from the same individuals. We found a significant correlation between rs11649743-G and elevated *HNF1B* levels in tumor tissue (*n* = 66) (*p* = 0.038) (Figure 2A), corresponding to the identification of the G-allele as the risk allele for PC [4]. In a smaller cohort of laser-captured micro-dissected prostate tumor tissue from 36 Danish patients, we found a significant correlation between risk SNP rs3760511-G [2] and elevated *HNF1B* levels (*p* = 0.018) (Figure 2B). These two SNPs are only in weak LD (Figure 1), and so these could represent distinct expression quantitative trait loci (eQTL) signals. Alternatively, they may interact at the chromatin level when *HNF1B* executes its functions as a transcription factor, and so together they could effectively constitute one signal. We found no association between rs4430796 (in LD with rs7501939; r^2^ = 0.764) and *HNF1B* expression levels, which may be due to our relatively small sample size compared to Grisanzio *et al*. (2012)[15], who identified a correlation between rs4430796 and *HNF1B* expression levels in benign tissues in three ethnic cohorts – European, Japanese and African Americans (total *n* = 407).

### *HNF1B* promoter methylation correlates with prostate cancer risk SNP genotype

**Figure 3:**
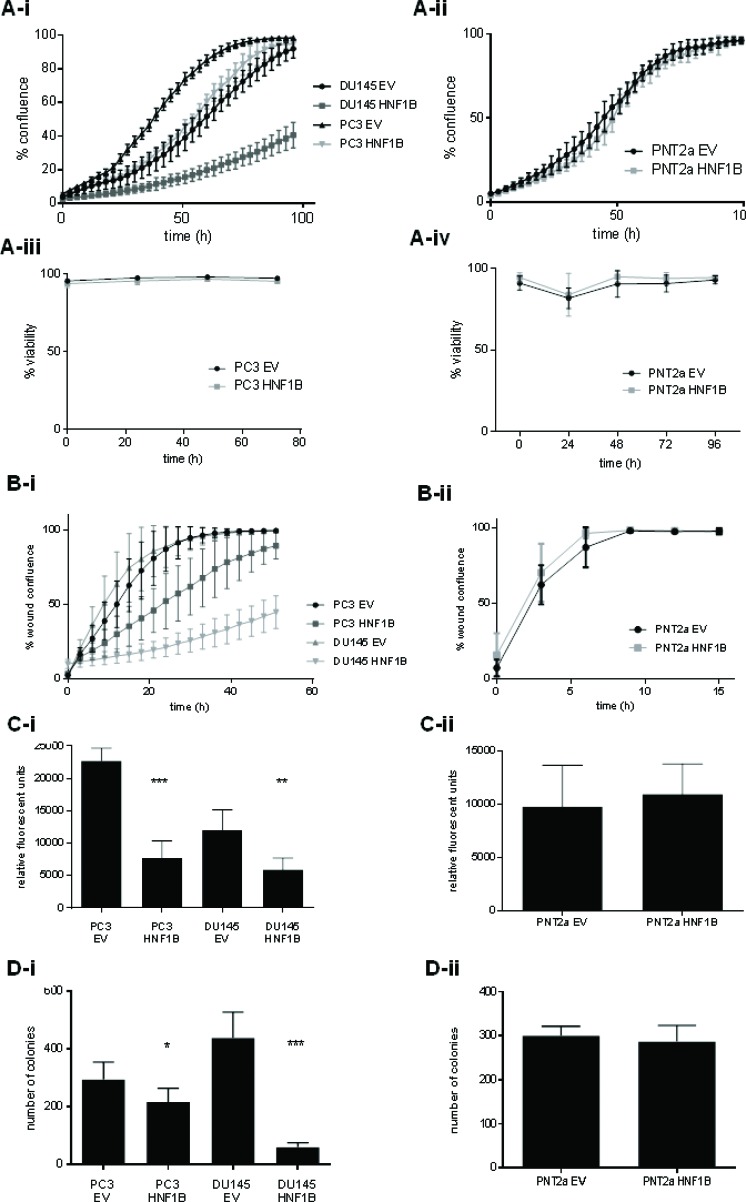
Methylation at the *HNF1B* promoter is significantly higher in prostate tumor tissues compared to matched benign in British (*t*-test *p* = 0.0076) (A) and Danish samples (B) (ranksum t-test; blue spots indicate statistical significance at the corresponding CpG probe) The significance threshold *p* < 0.05 (log_10_(p) > 1.301) is indicated by the green line. Ovarian cancer risk SNP rs757210 is also associated with *HNF1B* promoter methylation (pink spots) in 231 HGS cases (see also Supplementary Table 7). Histone modifications typical of active promoters – H3K4Me1, H3K4Me3 and H3K27Ac – overlap with the transcription start site, both small and large CpG islands and risk SNP rs3760511 (UCSC Genome browser, hg19). Figure 3Aiii. Increased methylation at *HNF1B* promoter correlates strongly with reduced levels of *HNF1B* expression prostate tissue (TCGA data). Pink spots = tumor; blue spots = matched normal tissue. Figure 3-Bi. Prostate cancer risk allele rs11649743-G is associated with reduced levels of *HNF1B* promoter methylation in tumor tissue, but not benign tissue in British samples (*p* = 0.0296), while risk allele rs3760511-G is associated with reduced levels of *HNF1B* methylation (*p* = 0.0283) in 21 pairs of Danish prostate cancer samples (3B-ii).

Promoter methylation plays a key role in transcriptional regulation, with the presence of methyl groups on cytosine bases at gene promoters essentially silencing gene expression. The *HNF1B* promoter harbors a CpG island that is a known target of epigenetic inactivation in high-grade serous (HGS) OC, as well as ovarian, colorectal, gastric and pancreatic cell lines [16, 17]. This CpG island has not previously been reported as a methylation target in PC (http://pubmeth.org). To determine whether epigenetic inactivation could play a role in regulating *HNF1B* expression levels in prostate cancer, we compared methylation levels at the *HNF1B* promoter between cancer and matched non-cancer samples in two cohorts (see Methods). Tumor tissues show significant hyper-methylation at this promoter compared to matched non-tumor tissues in both British (*n* = 59, *p* = 0.0076; Figure 3Ai) and Danish prostate cancer patients (*n* = 21, *p* = 0.0003; Figure 3Aii, blue dots). TCGA prostate cancer methylation data further confirm elevated HNF1B promoter methylation in tumor compared to normal tissue (Supplementary Figure 1). Hypermethylation at this promoter is also associated with reduced HNF1B expression in these same samples (Figure 3A-iii).

In addition, PC risk SNP rs11649743-G was associated with significantly lower levels of *HNF1B* promoter methylation in tumor tissues in British samples (*p* = 0.0296; Figure 3B-i), while promoter region risk allele rs3760511-G associated with reduced *HNF1B* promoter methylation in the Danish cohort of 21 pairs of laser-capture micro-dissected (LCM) tumor and normal and adjacent-normal samples (*p* = 0.0283; Figure 3B-ii).

Essentially, two established, independent PC risk alleles variants – rs11649743-G and rs3760511-G – are both associated with reduced promoter methylation at *HNF1B*, and simultaneously increased HNF1B expression in two different clinical prostate cohorts, suggesting a plausible mechanism for this observation. We found no association between any other PC risk SNP tested and promoter methylation in either cohort (Supplementary Figure 2A and 2B.).

### *HNF1B* has functional roles significant in prostate cancer

To identify biological pathways regulated by *HNF1B* in the context of cancer, we generated stable cell lines over-expressing *HNF1B* (see Methods). PC3 and DU145 are prostate cancer cells derived from bone and dura mater metastases (aggressive tumours), while PNT2a cells originate from normal prostate epithelial cells. Total RNA from PC3 cell line expressing empty vector (PC3-EV) and derived PC3-HNF1B cells was assayed on Illumina HT12 gene expression arrays, since this was the cell line with the highest over-expression of *HNF1B* (Supplementary Figure 3A-C). In total, 60 down- and 150 up-regulated genes were identified from two biological replicates, and used in subsequent analyses (Methods, Supplementary methods and Supplementary Table 1). Gene ontology (GO) enrichment analysis of the DEG following over-expression of *HNF1B* in this prostate cancer model suggested that biological pathways in cellular movement, growth and proliferation were over-represented (Supplementary Figure 4).

**Figure 4:**
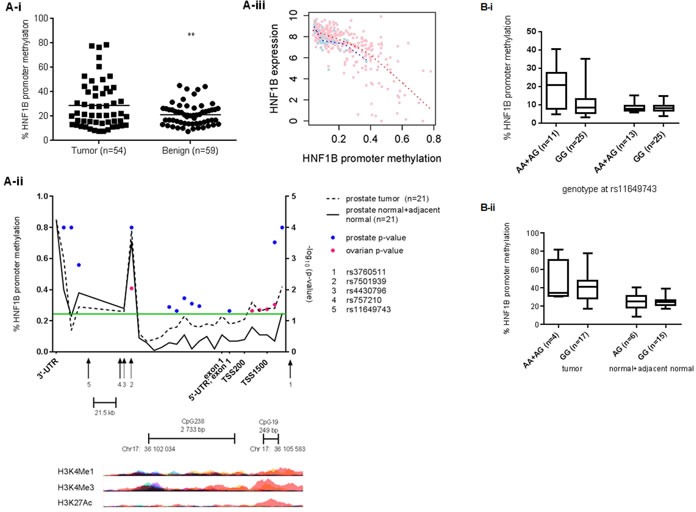
The differential effects of *HNF1B* in cancer and normal prostate cellular contexts Over-expression of *HNF1B* was associated with a reduction in rate of proliferation in prostate cancer lines PC3 and DU145 compared to empty vector (**4A-i**), but had no effect in normal PNT2a cells (4A-ii). No effect on viability on any cell line was observed (**4A-iii, iv**). *HNF1B* over-expression also correlated with a reduction in cell migration in PC3 and DU145 cells (*p* < 0.0001)(4B-i), but had no effect on cell migration in normal PNT2a prostate cells (p = 0.7771; **4B-ii**). The invasiveness of PC3 and DU145 cancer cells was also significantly reduced with over-expression of *HNF1B* (*p* < 0.0001 and *p* < 0.0003, respectively; Figure 4C-i). No difference was seen in normal PNT2a cells (*p* = 0.5358; Figure 4Cii). Clonogenic potential was significantly reduced in cancer models PC3 and DU145 over-expressing *HNF1B* (*p* = 0.01 and *p* < 0.0001 respectively) (Figure 4D-i), but showed no change in the PNT2a model (*p* = 0.2784, Figure 4D-ii).

Over-expression of *HNF1B* corresponded with a marked reduction in proliferation in PC3 and DU145 prostate cancer cells compared to empty vector (EV) controls (*p* < 0.0001, Figure 4A-i); no effect was observed in normal prostate PNT2a model (*p* = 0.6629; Figure 4A-ii), while viability was unaffected in all cell lines (Figures 4A-iii and 4A-iv) *HNF1B* over-expression also led to a decrease in the rate of cell migration in prostate cancer models (p < 0.0001) (Figure 4B-i), but *HNF1B* levels had no effect on cell migration in PNT2a cells (*p* = 0.7771; Figure 4B-ii). Invasiveness of normally highly metastatic PC3 and DU145 cells was significantly reduced on over-expression of *HNF1B* (*p* < 0.0001 and *p* < 0.0003, respectively) (Figure 4C-i), but *HNF1B* expression had no effect on the invasive potential of normal prostate cells (*p* = 0.5358; Figure 4-Cii). In addition, clonogenic potential was significantly reduced in PC3-HNF1B and DU145-HNF1B cells compared to EV (*p* = 0.01 and *p* < 0.0001 respectively) (Figure 4D-i), but was unaffected in PNT2a cells (*p* = 0.2784, Figure 4D-ii).

Over-expression of *HNF1B* also effected an obvious change in morphology. PC3-EV cells normally have an elongated mesenchymal morphology, but PC3-HNF1B cells acquired a flattened, epithelial-like morphology (Supplementary Figure 5A-C), an effect also observed in endometrial cells [17]. Paxillin is a major component of focal adhesion complexes and a ligand of integrin, and participates in cell adhesion-mediated signal transduction. As such, it has an important role in the regulation of cell shape and movement, and the morphological changes observed were associated with concomitant changes in the localization of the adaptor protein paxillin in these cells (Supplementary Figure 6A-F), as well as in DU145-HNF1B cells compared to DU145-EV controls (Supplementary Figure 6G-L). Neither cell line normally exhibits large peripheral adhesions; however, both PC3 and DU145 cells over-expressing *HNF1B* displayed large, prominent peripheral paxillin-associated adhesions. These changes were not evident in normal prostate PNT2a-EV or PNT2a-HNF1B cells (Supplementary Figure 6M-R), which both displayed very few paxillin-associated adhesions. A reduction in proliferation can affect some trans-well migration assays, but DU145 cells with larger paxillin-associated adhesions have been shown to migrate more slowly [18], so this is more likely to be the reason *HNF1B* over-expressing cells migrate less than their EV counterparts. The redistribution of paxillin into large peripheral adhesions was independent of total paxillin levels, which were comparable between PC3-EV and PC3-HNF1B, and DU145-EV and DU145-HNF1B cells (Supplementary Figure 7A and B).

The changes in localization of paxillin within the cell together with alterations in migration potential and prominent clustering at the cell periphery when *HNF1B* is over-expressed in prostate cancer models strongly suggest that loss of *HNF1B* expression has a fundamental role in EMT.

### *HNF1B*-related gene networks are enriched in clinical cancer studies

Using gene set enrichment analysis (GSEA)[19] we compared the 210-gene signature (Supplementary Table 1) associated with *HNF1B* over-expression *in vitro*, with gene expression data from five clinical prostate cancer studies, [20–24] to identify key genes related to functional phenotypes observed. The *HNF1B* gene signature was significantly enriched in four of five prostate cohorts (Supplementary Table 2 & Supplementary Figure 8A-D). Leading edge analysis in each cohort identified a subset of 129 key genes driving this enrichment (Supplementary Table 3). Pathway analysis suggested that chemotaxis and integrin- and cadherin-mediated cell adhesion were the most functionally relevant processes (GeneGo Metacore; Supplementary Figure 9), with two genes present in every sample type comparison (e.g. benign vs. tumor) across all studies - *FLRT3* and *SLC14A1*.

Both targets are co-expressed with *HNF1B* in clinical prostate samples, and in each case we found elevated levels associated with relapse-free survival (*p* = 0.00025 and *p* = 0.00026, respectively) (Supplementary Figure 10 A-B) [21]. This mirrors the association reported by Glinsky *et al*., (2004), where increased levels of HNF1B were significantly associated with improved prognosis (*p* = 0.0093) in a primary prostate tumor expression data set (Supplementary Figure 10 C) [24]. mRNA levels of *FLRT3* and *SLC14A1* also showed a stepwise reduction from benign to primary prostate tumor and metastatic samples in other clinical prostate datasets (Supplementary Figure 11)[20–22, 25].

### Mechanistic role of *HNF1B*

Using quantitative RT-PCR, we subsequently confirmed over-expression of novel targets *FLRT3* and *SLC14A1* and two known *HNF1B* target genes [26] in PC3-HNF1B and DU145-HNF1B cells, but not in PNT2a cells despite significant over-expression of *HNF1B* in this model (Supplementary Figure 12 A-E). Finally, chromatin immunoprecipitation (ChIP) for HNF1B in PC3-HNF1B cells followed by qPCR also showed enrichment of *FLRT3* and *SLC14A1*, indicating a direct interaction between HNF1B transcription factor at these target genes at the chromatin level (Supplementary Figure 13). This suggests that epigenetic inactivation of *HNF1B* could bring about the phenotypic changes observed in the cell-based functional assays via these genes.

*FLRT3* is a member of the fibronectin leucine-rich transmembrane protein family, is expressed at the cell surface and has an established role in the regulation of FGF receptor signaling [27] and cadherin-mediated cell adhesion and morphogenesis [28, 29]. Our functional and bioinformatic analyses support this, and suggest that candidates associated with these processes in particular may be relevant to the effects of *HNF1B* in a cancer context. SNP variants within *SLC14A1* have been associated with urinary bladder cancer risk [30], and identified as a potential biomarker in prostate cancer [31]. *SLC14A1* encodes urea transporter B (UT-B), which facilitates the clearance of urea (metabolized from L-arginine) from cells. The loss of this transporter in bladder results in the accumulation of urea and reduced expression of arginase I [32], with a concomitant reduction in levels of ornithine and polyamines, and an increase in nitric oxide (NO) production. This makes cells vulnerable to DNA damage, with DNA-damaging reactive NO up-regulated [32] and DNA-stabilising polyamines down-regulated [33]. Interestingly, an eQTL at prostate cancer risk locus SLC22A3 (encoding polyamine transporter OCT-3) was recently reported [15], with the PC risk allele associated with lower levels of SLC22A3 in prostate tissues, and reduced viability *in vitro* [15].

### *HNF1B* has similar functions and mechanism in ovarian cancer

*HNF1B* is part of a five-gene expression signature predictive of relapse in PC patients [24], and is also a biomarker that can distinguish clear cell OC from other subtypes[34]. Different SNPs in *HNF1B* are associated with each disease [9, 17, 35] and different subtypes of OC [9, 17, 36]. Since the rs757210 OC risk-associated SNP is in LD with key prostate cancer risk SNPs (r^2^ = 0.739 with rs7501939; r^2^ = 0.543 with rs4430796) (Table 1), we tested whether we could re-capitulate our key findings in an ovarian context.

Firstly, we found similar pathways relating to extra-cellular matrix re-modelling and cellular adhesion enriched in an OC model, following shRNA knock-down of *HNF1B* in RMG2 ovarian cancer cells (GEO, GSE37290) (Supplementary Figure 14 & Methods). In addition, the 210-gene signature associated with *HNF1B* over-expression in prostate was enriched in two clinical OC cohorts [37] [38] (Supplementary Figure 15), where *FLRT3* was identified in the leading edge gene set in each case (Supplementary Tables 4 & 5). *SLC14A1* was only marginally significant.

We compared the prostate (129) and ovarian (45) leading edge gene, and identified 37 genes common to both diseases (Supplementary Figure 16A; Supplementary Table 6). Chemotaxis and cadherin-mediated adhesion to ECM are key biological processes related to this gene set (Supplementary Figure 16B), which is altered (amplified/deleted/mutated) in 72% of 246 PC cases and 82% 316 OC cases, based on The Cancer Genome Atlas (TCGA) data. None is a known cancer gene (http://cancer.sanger.ac.uk/cancergenome/projects/cosmic/). FLRT3 is most strongly co-expressed with tumor suppressor gene *TP63* and angiogenesis regulator gene *NTN4* (Pearson correlation r = 0.83) (CBioportal), which suggests possible mechanisms by which *HNF1B* could exert the effects observed here – a control switch preventing EMT in non-tumor tissue.

We have shown that *HNF1B* promoter methylation in PC is associated with known PC risk SNPs (Figure 2). Using 450K methylation array data from the Mayo Clinic [39, 40], we found a significant association between a linked OC risk SNP in *HNF1B* rs757210, and tumor methylation in *n* = 231 high-grade serous OC (Figure 3B pink dots, and Supplementary Table 7). Although this specific variant was not associated with *HNF1B* expression levels in 182 HGS ovarian cancer samples (*p* = 0.2032) (Supplementary Table 8A), it was borderline significant in a much smaller set of 21 clear cell ovarian cancer patients (*p* = 0.0732) (Supplementary Table 8B) based on gene expression array data from samples genotyped as part of the Collaborative Oncological Gene-environment Study (COGS)[9]. However, we did identify a significant association between this SNP rs757210 and tumor methylation in HGS OC (Figure 3A and Supplementary Table 7), consistent with the report of Pharoah *et al*. (2013). TCGA data and fine-mapping of the region has previously identified two loci 6,8 kb apart, associated with increased promoter methylation in high-grade serous cases (rs7405776) and increased *HNF1B* expression in clear cell OC cases (rs11651755)[17], confirming our results: both of these SNPs - independent of each other - correlate with rs757210 (r^2^ = 0.6 and r^2^ = 0.97, respectively), which lies half-way between them. Furthermore, rs11651755 is also in strong LD with PC risk SNP rs4430796 (r^2^ = 0.97).

There is an obvious overlap between regions of methylation within *HNF1B* in prostate and ovarian cancer tissues, with a methylation signal at the 3'-UTR in both. However, the most significant methylation occurs upstream of the TSS at the small CpG: 19 island and coincides with poised and active enhancers, identified by the presence of H3K27Ac, H3K4Me1 and H3K3Me3 histone marks [41](Figure 3Aii). Poised enhancers can be activated during differentiation or in response to external stimuli, and *HNF1B* is known to be important in embryonic urogenital development, where its role in EMT– as suggested by the sum of our functional data here – would be appropriate. However, if this role were disrupted by stressors typical of tumor cells, such as aberrant metabolism (a hallmark of cancer), *HNF1B* would then be tumor promoting. Indeed, the deregulation of pathways that maintain quiescence of ovarian surface epithelial cells has been shown to be instrumental in the progression to serous ovarian cancer [38], and would present this sort of insult. A similar effect has also been observed in renal cell carcinoma, where *HNF1B* expression correlated with malignant transformation and progression, with elevated levels of *HNF1B* expression in primary tumor associated with better prognosis [42]. Our findings highlight the multiple, small blocks of linkage disequilibrium within *HNF1B*, and may explain the complex associations observed at this locus between different risk SNP alleles, *HNF1B* expression and promoter methylation depending on cancer or histological subtype.

**Figure 5:**
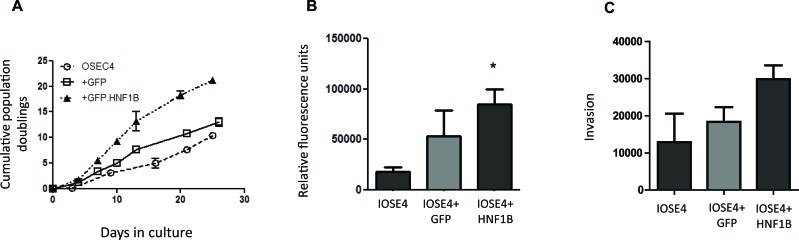
Over-expression of *HNF1B* in ovarian IOSE4 cells is associated with increased proliferation (5A), migration (5B) and invasion (5C) compared to IOSE4 cells expressing control GFP

Finally, to assess the phenotypic effects of *HNF1B* in the context of ovarian cancer, we over-expressed *HNF1B* in IOSE4 ovarian epithelial cells and tested typical tumor phenotypes: we observed significant effects on proliferation (Figure 5A), migration (5B) and invasiveness (Figure 5C) in IOSE4-HNF1B cells compared to IOSE4-EV control cells. There is considerable evidence to indicate that *HNF1B* is over-expressed and behaves like an oncogene in clear cell OC [43], but is lost and acts as a tumor suppressor in HGS OC [44, 45]. Our data further support this. Similarly, our data suggests that that *HNF1B* may act as a tumor suppressor in benign prostate tissue, where it works normally to suppress classic features of tumorigenesis, by stimulating transcription of genes with clear roles in controlling cellular proliferation, adhesion and movement. On inactivation by DNA methylation in the progression to more aggressive tumors, these protective effects are lost.

## DISCUSSION

*HNF1B* appears to play a significant role in the etiology and early stage neoplastic development of both prostate and ovarian cancers. We link intronic SNPs with *HNF1B* expression levels and epigenetic silencing in multiple populations in both prostate and ovarian cancers, thereby suggesting a plausible mechanism of action. In addition, our experimental data show that similar mechanisms contribute to prostate and serous ovarian cancer development and progression, and demonstrate the value of cross-site cancer analyses to functionally validate consistent GWAS findings across different disease types. In a clinical setting, the implication is that individuals carrying the high-risk genetic variants (rs11649743-G and rs3760511-G in prostate cancer; rs757210-G in serous ovarian cancer) are primed for EMT, which could be driven by additional stressors typical of tumor cells, such as aberrant metabolism – a hallmark of cancer. Most importantly, both increased transcript expression levels and reduced degree of promoter methylation are tagged by the same easily-screened genetic markers. The independent expression-methylation quantitative trait locus (eQTL-mQTL) associations identified here support the idea of pleiotropy as a common functional mechanism underlying the biology of neoplastic development at risk loci identified by GWAS. More comprehensive whole-genome eQTL-mQTL investigations may be informative for *HNF1B* specifically, as well as more generally for (epi)genome-wide association studies (EWAS/GWAS) This finding further emphasizes the usefulness of considering disease mechanisms across distinct cancer types to describe a conserved and significant general mechanism of action.

## MATERIALS AND METHODS

### Patient samples

British prostate cancer and matched benign samples were taken from patients recruited under the Prostate Mechanisms for Progression and Treatment (ProMPT) study MREC 01/4/061, and have been previously described [46]. Ovarian cancer samples were taken from patients recruited into the OCAC consortium[9] at the Mayo Clinic, under approval of the Mayo Clinic Institutional Review, and have been previously described [9] [39, 40]. Danish prostate cancer and matched normal and adjacent normal samples were from patients recruited at Aarhus university hospital, under approval of the Aarhus University Regional Ethical committee and the Danish Data Protection Agency.

All participants at each of the three study sites (Cambridge, Aarhus, Mayo Clinic) provided written informed consent; all experimental protocols at each of the three sites were approved by named local research ethics committees. All testing was undertaken in accordance with local, approved guidelines and regulations at each site.

### Cell culture

PC3, DU145, and PNT2a cell lines were obtained from ATCC, and grown in RPMI supplemented with 10% FBS (Invitrogen) at 37C, 5% CO_2_. Stable PC3-HNF1B and DU145-HNF1B cell lines were grown under selection of 100 μg/ml geneticin (Gibco); PNT2a cells were grown under selection of 2 μg/ml puromycin (Invitrogen). IOSE4 cells were grown under selection of 400 ng/ml puromycin.

### Stable cell lines over-expressing HNF1B

PC3 and DU145 cells were transfected with a modified rc/CMV vector to over-express *HNF1B*, to generate stable cell lines PC3-HNF1B and DU145-HNF1B. PNT2a-HNF1B and OSE4-HNF1B cells were generated as described [17]. Parental cells were transfected with the corresponding empty vector (EV) or GFP as a control.

### siRNA

Cells were transfected with 50 nM siRNA with RNAi Max (Invitrogen) by reverse transfection, according to manufacturer's instructions. *HNF1B* was silenced using 50 mM siRNAs SASI_Hs02_00302585 and SASI_Hs02_00302586 in combination (Sigma). Universal negative control #1 (SIC001, Sigma) was used as a negative control.

### Western blots

Whole cell lysates were run on 4-16% SDS-PAGE gels (ThermoFisher) and transferred to PVDF membranes (Invitrogen). Antibodies used were: anti-HNF1B (H-85) (SC-22840, Santa Cruz), anti-B-actin (AC-15) (ab6276, Abcam), anti-paxillin [Y-113] (ab32084, Abcam), anti-integrin α2 (611016, BB Biosciences), anti-integrin β1 (610467, BD Biosciences).

### Genotyping

British prostate samples were genotyped as part of the PRACTICAL consortium on custom SNP Illumina arrays (iCOGS)[47]. Danish prostate samples were genotyped using commercial TaqMan assays C___2559918_10 (rs11649743), C___2559889_10 (rs4430796), C___2960803_10 (rs7501939) and C__26657407_10 (rs3760511). Ovarian samples were genotyped as part of the OCAC consortium using Illumina infinium iSelect BeadChips and iCOGS arrays[9].

### Gene expression microarrays

Cell lines: Total RNA was harvested from 2 biological replicates (4 technical replicates each) of PC3-EV and PC3-HNF1B cells (RiboPure, Ambion). UK clinical material: Total RNA was extracted from prostate tissue sections from fresh-frozen radical prostatectomy biopsies (AllPrep, Qiagen). Tumor or non-tumor was selected by pathologist A.Y.W. Danish clinical material: normal and cancer tissue sections were laser capture micro-dissected, RNA extracted and assayed on Affymetrix U133A gene expression arrays. See also Supplementary Methods.

### Promoter methylation analysis

British prostate samples: gDNA was extracted from fresh, frozen radical prostatectomy tissue (AllPrep, Qiagen). Unmethylated cytosine bases were sodium bisulfite converted with EpiTect Bisulfite kits (Qiagen). Converted gDNA was amplified at *HNF1B* promoter CpG islands using assay PM00178808 and PyroMark® kits (Qiagen). Methylation levels at each amplicon were determined using Pyrosequencing – see Supplementary Methods. Danish prostate samples: gDNA from 21 Danish patient prostate cancer tumor samples (T), 12 adjacent non-malignant prostate tissue (AN) and 9 normal prostate tissue (N) from patients with bladder cancer but no prostate cancer were assayed on Illumina 450K methylation arrays. Mayo Clinic ovarian cancer sample data were from 450K methylation arrays, generated as previously described [39, 40].

### Chromatin immunoprecipitation

ChIP reactions were performed as described previously [48], using anti-HNF1B antibody (H-85) (SC-22840, Santa Cruz.

### Data access

*in vitro* HNF1B over-expression gene expression assay data are entered at GEO under GSE63134.

## SUPPLEMENTARY MATERIALS FIGURES AND TABLES




