# Supplementary material for: HNF1B variants associate with promoter methylation and regulate gene networks activated in prostate and ovarian cancer
Source: Oncotarget. 2016 Oct 9;7(46):74734–46. doi: 10.18632/oncotarget.12543 (PMC5342698; doi:10.18632/oncotarget.12543)
Supplement: Supplementary file 1 [file oncotarget-07-74734-s001.pdf]

HNF1B variants associate with promoter methylation and regulate gene networks activated in prostate and ovarian cancer

Supplementary Materials

Cell culture

Cell lines were authenticated by STR profiling before work commenced, and tested for *Mycoplasma* infection monthly by ELISA assay.

Gene expression microarrays

For all samples, Illumina BeadChip (HumanWG 12 version 3) bead-level data were preprocessed to remove spatial artifacts, log2-transformed and quantile normalized using the *beadarray* (16) package in Bioconductor prior to analysis. Differential gene expression analysis was performed in *limma* package using the empirical Bayes method, and the Benjamini & Hochberg false discovery rate method (adjusted p-value<0.01)(17) was used to correct for multiple testing. In the PC3-HNF1B *in vitro* model, 4,547 differentially expressed genes (DEG) were identified in four biological replicates (false discovery rate (FDR) p<0.01). Only statistically significant genes also showing a ≥3-fold change in gene expression were considered in subsequent analyses; a total of 60 down- and 150 up-regulated genes (log fold change +/- 1.5) were identified (Supplementary Table 1).

Danish clinical material: Data were log2 normalised. Mayo Clinic ovarian cancer samples (n=182 HGS and n=21 CC) were assayed using Agilent gene expression arrays(18,19). Publicly-available pre-processed Affymetrix Human genome U133A plus 2.0 gene expression data from *HNF1B* shRNA knock-down RMG2 ovarian cancer cell lines and non-targeting siRNA controls were downloaded from GEO (accession GSE37290); data were analysed in GEO2R (http://www.ncbi.nlm.nih.gov/geo/geo2r/).

Promoter methylation analysis

PCR products were hybridized to streptavidin Sepharose™ high-performance beads (GE Healthcare). Bead-bound product was then rinsed in 70% ethanol, 0.2M NaOH and 1X wash buffer (Qiagen), before being combined with 0.3 µM sequencing primer. Data were captured using qCpG software (Biotage). Danish prostate samples: Data along the length of *HNF1B* gene were averaged, and the mean at each locus compared (T vs N+AN) by ranksum t-test; p<0.05 ($-\log_{10}p>1.301$) was considered significant.

Pathway analysis

Gene ontology pathway enrichment was performed using GeneGo MetaCore (Thomas Reuters) and Ingenuity Pathway Analysis using default settings and FDR $p \leq 0.05$.

Gene set enrichment analysis

This was performed in GSEA (www.broadinstitute.org/gsea) with 'GSEAPreranked', using default settings and 1000 permutations. Data from publicly-available prostate and ovarian cancer gene expression studies were ranked according to t-statistic; sample contrasts are described in Supplementary Table 2. Profiles for the running enrichment scores – the degree to which the *HNF1B* gene signature set was overrepresented at the top or bottom of the expression dataset of the clinical study tested, and the position of each of the 210 HNF1B-DEG within each clinical study's rank ordered list (rank metric score) were generated (Supplementary Tables 3-5). The key genes contributing to the 'leading edge' are highlighted.

Quantitative PCR

For cell-based experiments, qPCRs were performed using 2X SYBR® Green master mix (Invitrogen) on ABI 9700 thermal cyclers (Applied Biosystems) using default settings. Data were analyzed using the ΔΔCt method. qPCR assays on clinical material were performed using the Fluidigm Biomark™ HD system with 96x96 Dynamic Arrays according to manufacturer's instructions. TaqMan assays Hs01001602_m1 (*HNF1B*), 4319413E (18S RNA), 4326315E (B-actin) and 4326314E (*RPLPO*) were used. Each assay was performed in triplicate, and two replicate plates were run for each assay. Data were normalized to the least variable housekeeping gene. Control RNAs (Clontech qPCR human reference total RNA, Cat No. 636690, Ambion FirstChoice human brain RNA reference, Cat No. AM6050; Applied Biosystems TaqMan control total RNA (human) Cat No. 4307281) were used as positive controls and to generate a scaling factor for accurate comparison of samples.

Genotype and gene expression correlation

British samples: Kruskal-Wallis (KW) tests were performed 1000 times for every correlation of genotype and gene expression level. The median of the p-values was taken as the final p-value (significant $p<0.05$). Danish samples: log2 normalized expression data (probe 205313_at) were correlated with genotype, with KW $p<0.05$ significant.

In vitro functional assays

Proliferation and cell viability

Equal numbers of cells were seeded into 6-well plates; conditions were assayed in triplicate. Proliferation was assayed using percentage cell confluence as a proxy for proliferation rate in the IncuCyte live cell analysis system (Essen Bioscience), according to manufacturer's instructions. For viability, for each condition at each time point (0h, 24h, 48h, 72h, 96h, 120h), supernatants and trypsinized cells were combined and counted using Trypan blue staining and a Beckman Coulter ViCell haemocytometer.

Invasion

Assays were performed in multiples of 8 for each condition using CytoCelect™ 96-well Cell Migration and Invasion (8µm, fluorometric format) plates (CBA-106, Cell Biolabs, Inc.), according to manufacturer's instructions.

Migration

Cells were grown to 80% confluency in full-serum media in 24-well ImageLock microplates (Essen Bioscience) in replicates of 8 for each condition. Cells were serum-starved for a minimum of 24h, or until 100% confluent. A fixed-width scratch was made in the cell monolayer with an 8-pin WoundMaker (EssenBioscience). Media was aspirated, and the cell monolayer washed twice with 1X PBS. Appropriate full-serum media was applied before cells were incubated at 37°C in an Incucyte Live Cell Imaging system. Image data were captured every hour. Custom algorithms calculated the 'Relative Wound Density' over time for each condition

Colony formation

6-well plates were seeded with 1000 cells per well and incubated for 10 days. Media was changed every 4 days. Cell were fixed with 1:1 acetone:methanol (10 min), stained with Giemsa stain (10 min), then air dried. Colonies were counted using a GelCount™ (Oxford Optronics).

Elongation

Cells were cultured on acid -washed cover slips, fixed with 3.7% paraformaldehyde and permeablilized with 0.1% Triton-X. Non-specific labeling was blocked with 3% BSA prior to incubation with paxillin primary antibody (BD Biosciences) or TRITC-phalloidin (red). Primary antibody was labeled with FITC (green). Cells were imaged using a Nikon Eclipse TS100 microscope at 20X magnification.

Confocal microscopy

Cells were grown in 8-well ibidi microscopy chambers (Gmbh), formaldehyde-fixed (4%), permeabilized in 0.1% Triton-X 100. Non-specific labeling was blocked with Odyssey blocking buffer (Licor, 927040000) for 30 min at room temperature. Actin was stained with phalloidin-TRITC (Sigma), primary antibodies anti-paxillin Y-113 (abcam, ab32084), integrin α2 and β1 (BD Biosciences kit, 611435) were diluted in blocking buffer at 1:200, 1:100 and 1:100 respectively. Alexa Fluor®-conjugated secondary antibodies were used at 1:1000 (anti-rabbit 488 IgG (H+L) and anti-mouse 647 IgG (H+L), in blocking buffer. Nuclei were stained with 10 µg/ml Hoechst 33342 trihydrochloride, trichlorate (Invitrogen, H3570). Cells were imaged using a Leica SP2 confocal microscope and acquired images processed using Leica LCS Light software.

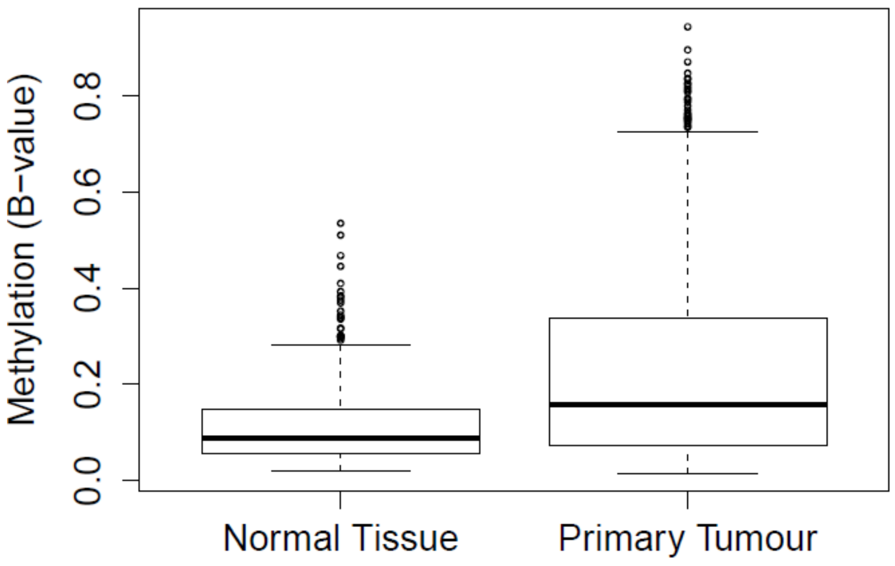

Supplementary Figure 1. TCGA data shows increased methylation at the *HNF1B* promoter in primary prostate tissue compared to matched normal.

A

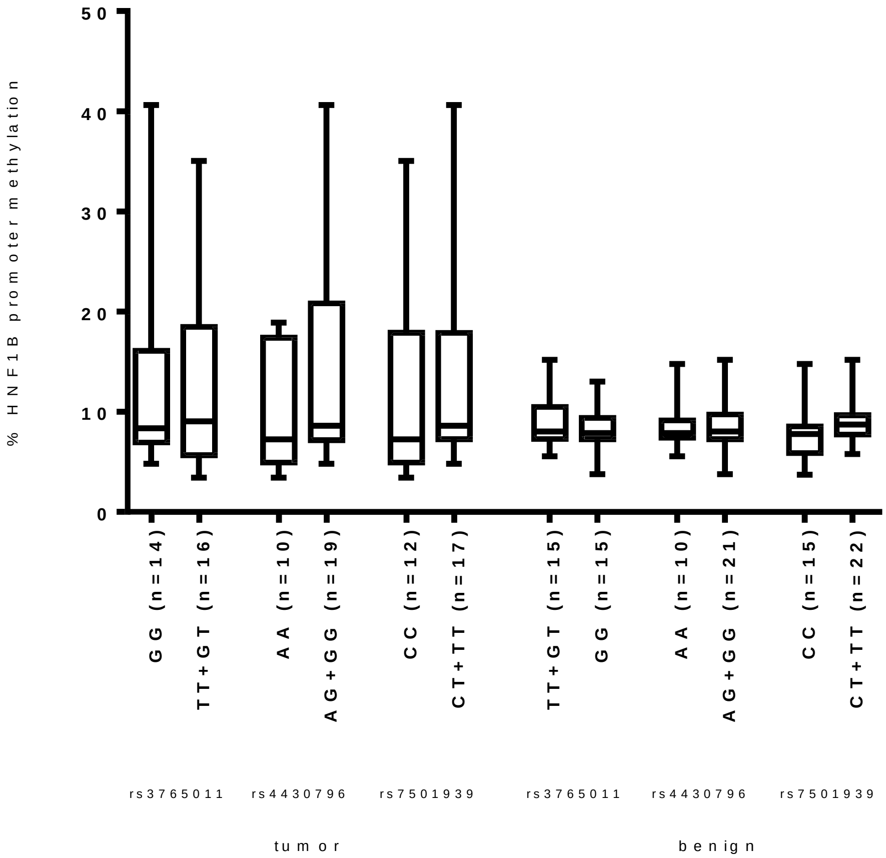

% HNF1B promoter methylation
50
40
30
20
10
0
GG (n=14)
TT+GT (n=16)
AA (n=10)
AG+GG (n=19)
CC (n=12)
CT+TT (n=17)
TT+GT (n=15)
GG (n=15)
AA (n=10)
AG+GG (n=21)
CC (n=15)
CT+TT (n=22)
rs3765011
rs4430796
rs7501939
rs3765011
rs4430796
rs7501939
tumor
benign

B

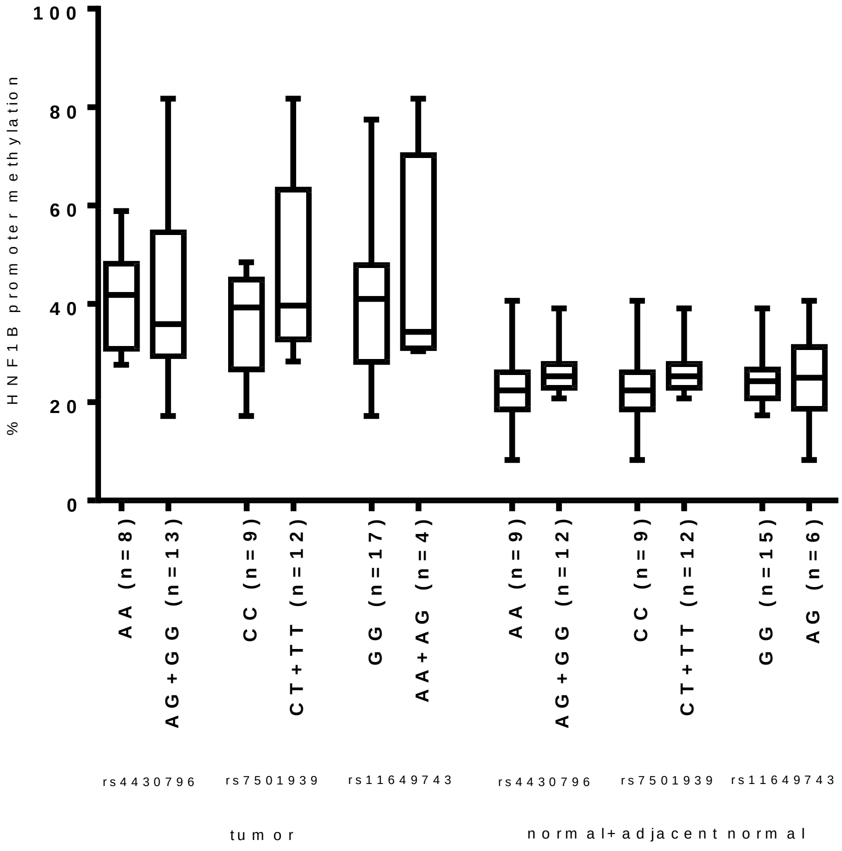

% HNF1B promoter methylation
100
80
60
40
20
0
AA (n=8)
AG+GG (n=13)
CC (n=9)
CT+TT (n=12)
GG (n=17)
AA+AG (n=4)
AA (n=9)
AG+GG (n=12)
CC (n=9)
CT+TT (n=12)
GG (n=15)
AG (n=6)
rs4430796
rs7501939
rs11649743
rs4430796
rs7501939
rs11649743
tumor
normal+adjacent normal

Supplementary Figure 2. There is no correlation between genotype and *HNF1B* promoter methylation levels at other key GWAS SNPs rs7501939 or rs4430796 in tumour or benign tissues in either British (A) or Danish (B) cohorts.

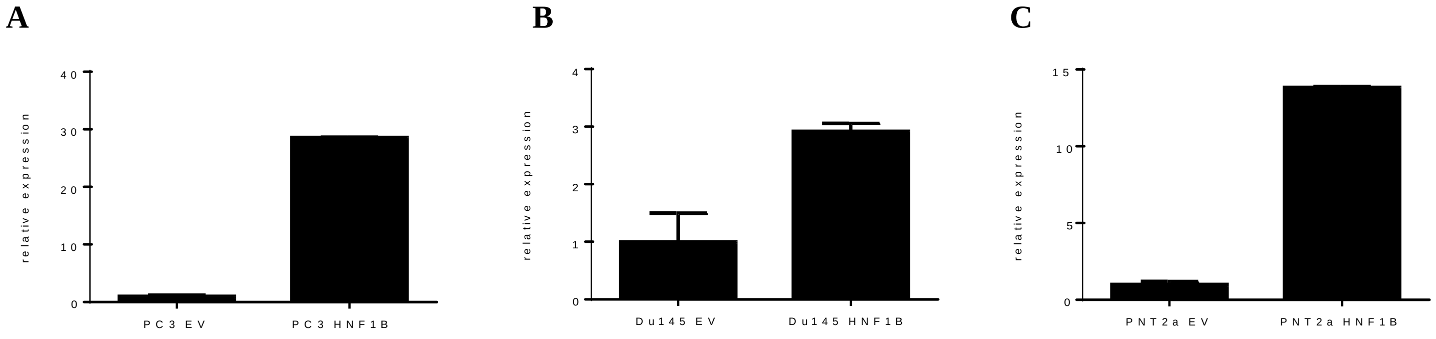

Supplementary Figure 3. Stable cell lines over-expressing *HNF1B* were derived for prostate cancer (**A, B**)and normal (**C**) cells. qRT-PCR confirmed over-expression of *HNF1B* in transfected cells compared to cells expressing an empty vector (EV). Data were normalized to actin.

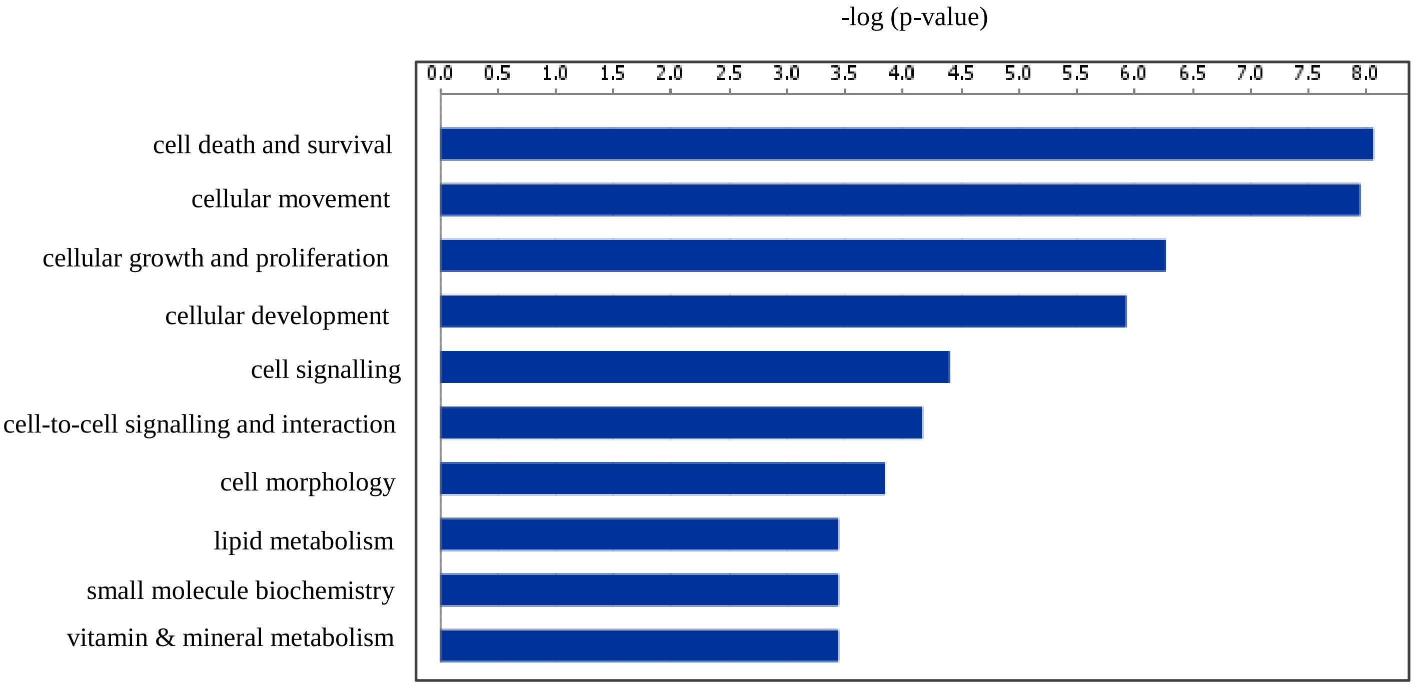

Supplementary Figure 4. Over-expression of *HNF1B* is associated with biological pathways involved in cellular movement, growth and proliferation. Gene expression profiling of PC3 EV vs. PC3 HNF1B cell lines identified a total of 4, 547 DEGs (FDR p<0.01) were identified from two biological replicates performed in triplicate. Gene ontology enrichment analysis was performed in Ingenuity Pathway Analysis software, using only statistically significant genes also showing a ≥3-fold change in gene expression (log FC ± 1.5); a total of 210 genes (60 down-and 150 up-regulated).

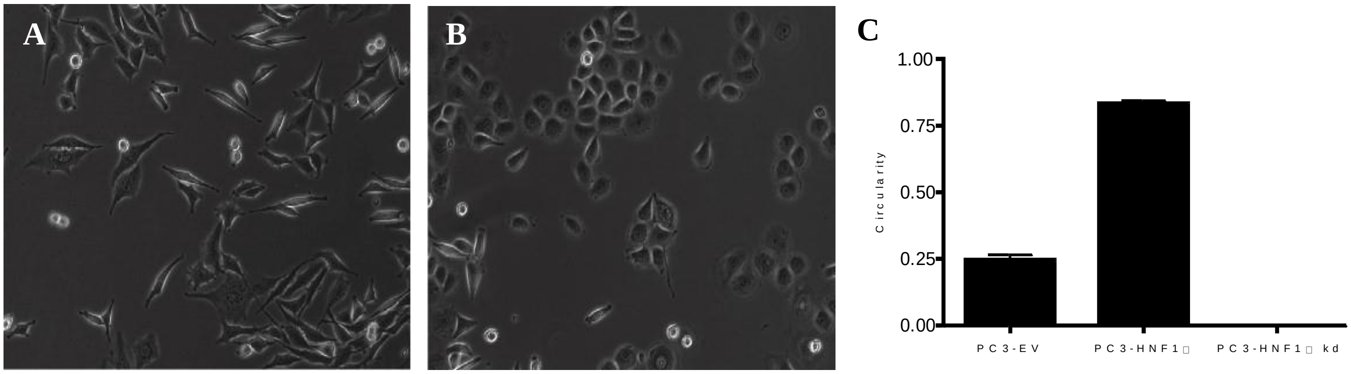

Supplementary Figure 5. Over-expression of *HNF1B* in PC3 produces a clear change in cell morphology between spidle-shaped PC3 EV cells (**A**) and more epithelial-like PC3
HNF1B cells (**B, C**).

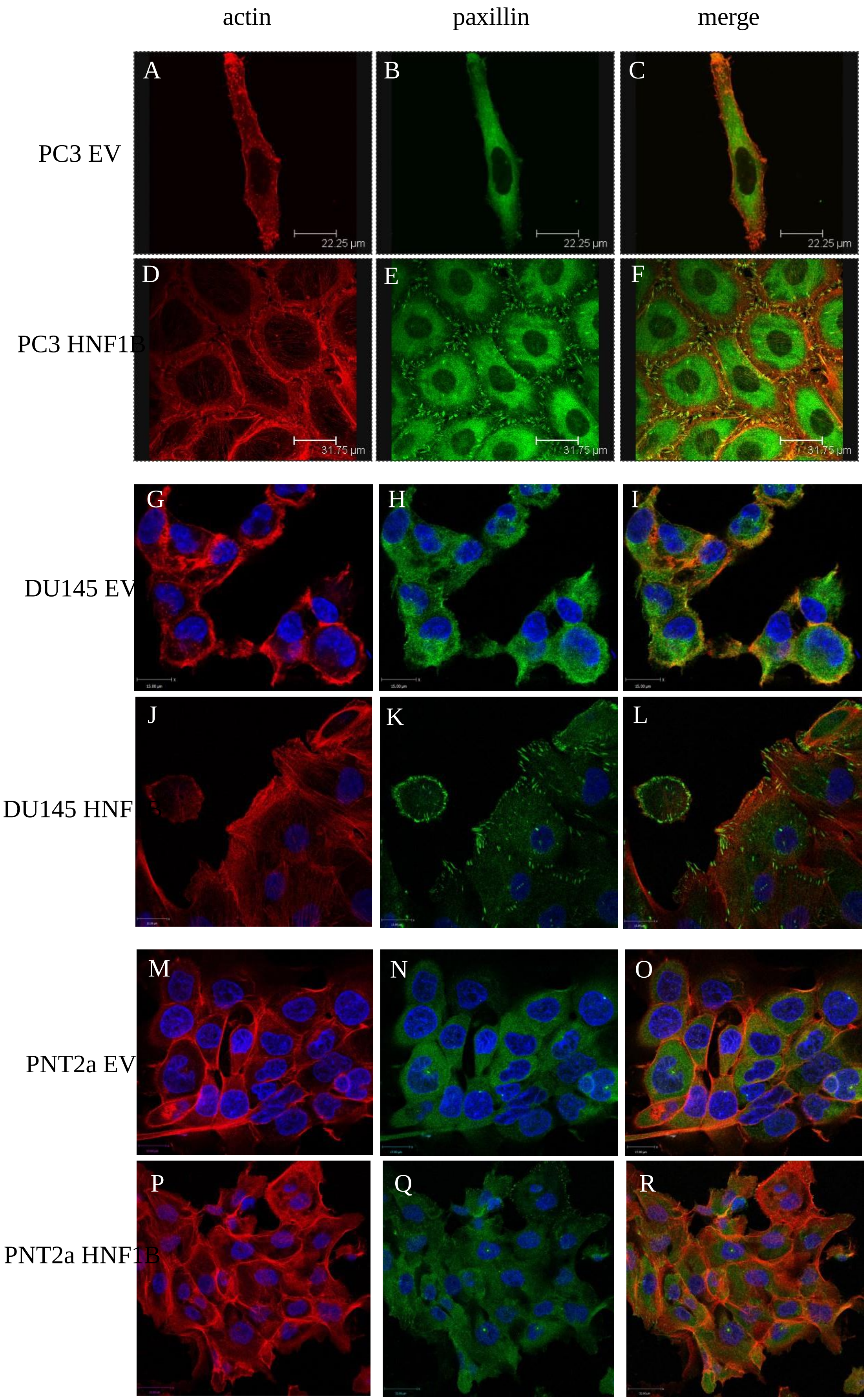
actin
paxillin
merge
PC3 EV
PC3 HNF1B
DU145 EV
DU145 HNF
PNT2a EV
PNT2a HNF1B
A
B
C
D
E
F
G
H
I
J
K
L
M
N
O
P
Q
R
22.25 µm
31.75 µm

Supplementary Figure 6.

Over-expression of *HNF1B* is associated with changes in the localization of the adaptor protein paxillin (green) in PC3 cells (**A-C**) compared to PC3 HNF1B (**D-F**), as well as in DU145 EV cells (**G-I**) compared to DU145 HNF1B (**J-L**). Neither cell line normally exhibits large peripheral adhesions; however, both PC3 and DU145 cells over-expressing *HNF1B* displayed large, prominent peripheral paxillin-associated adhesions. These changes in cell shape and distribution of paxillin were not evident in normal prostate PNT2a cells over-expressing *HNF1B* (**M-R**). Actin cytoskeleton is stained red (TRITC-phalloidin); nuclei are stained blue (DAPI).

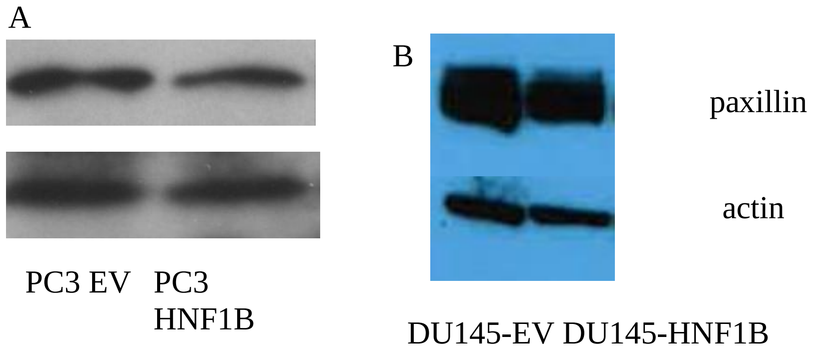

Supplementary Fig 7. Total levels of paxillin are unaffected by *HNF1B* levels in prostate cancer lines

Lysates (30 µg) from cells expressing empty vector (EV) or *HNF1B* were separated by SDS-PAGE. Proteins were transferred onto a PVDF membrane and probed with anti-paxillin and anti-beta actin antibodies. No differences in total paxillin levels were observed in PC3 (**A**) or DU145 (**B**).

A

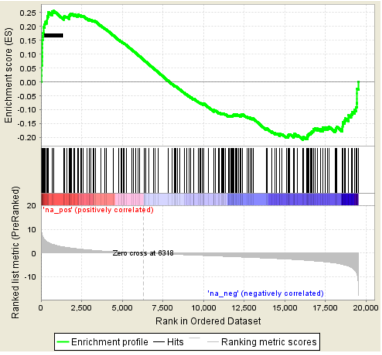

benign-primary PC; NES 1.36, FWER p <0.001

B

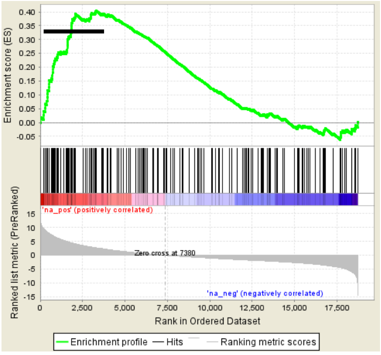

normal-primary PC; NES 1.83, FWER p<0.001

C

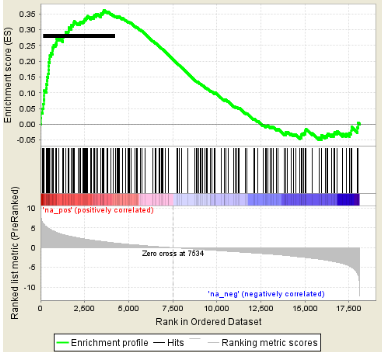

benign-primary PC; NES 1.78, FWER p<0.001

D

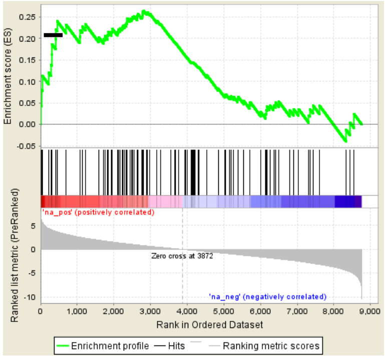

normal-primary PC; NES 1.25, FWER p=0.015

Supplementary Fig 8. A comparison of 210 *HNF1B*-related genes with ranked gene expression data from publically available PC data sets showed significant enrichment of our gene set in four clinical prostate cancer studies: (A) Varambally *et al*., (2005), (B) Taylor *et al*., (2010), (C) Grasso *et al*., (2012) and (D) Tomlins *et al*. (2007) [1-4]. Defined subsets of genes are considered enriched in a larger set if their distribution is not random throughout, but skewed towards either the top or bottom of the distribution, suggesting genes related to the phenotypes observed. Leading edge analysis (thick black bars) identified the gene set driving enrichment in each comparison. A consensus set of 129 genes was identified by combining all comparisons. Pathway analysis on this subset revealed an enrichment of genes relating to cadherin-mediated adhesion and epithelial-mesenchymal transition (EMT). For all comparisons performed, see Supplementary Table 3.

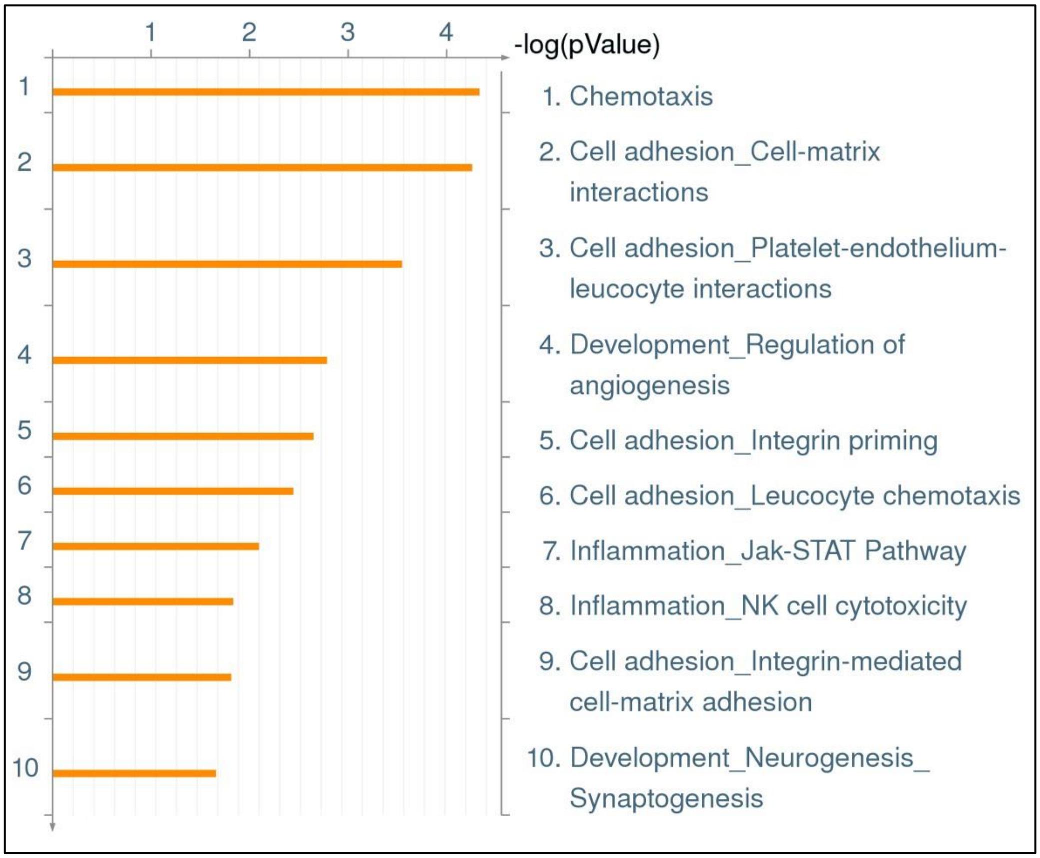

Supplementary Fig 9. Pathway analysis (GeneGo Metacore) of the 129 ‘leading edge’ genes across all clinical prostate studies, identified cellular adhesion and chemotaxis as significant networks enriched in this gene set.

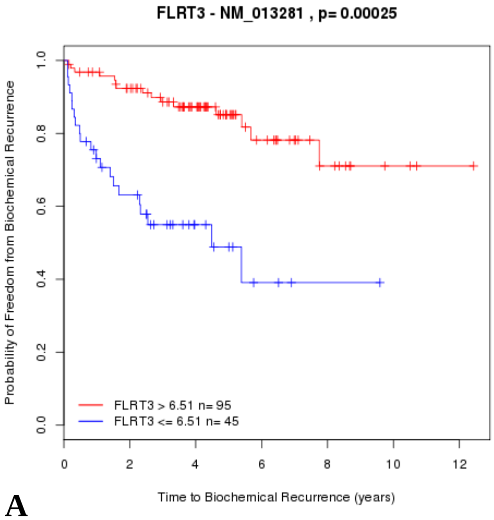

A

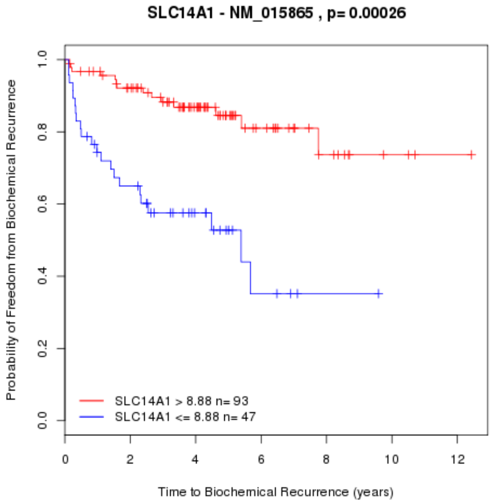

B

C

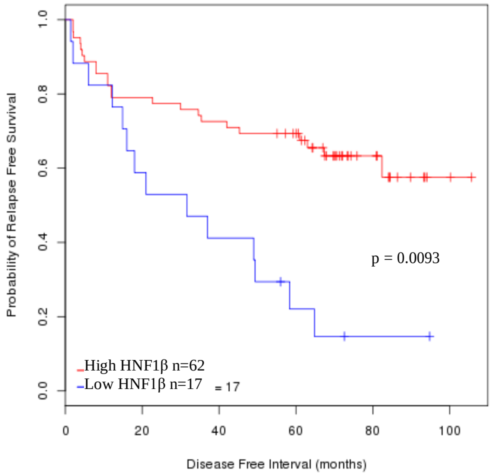

Supplementary Fig 10. Elevated levels of *FLRT3* (**A**) and *SLC14A1* (**B**) are associated with significantly improved odds of relapse-free survival. Kaplan-Meier curves and recursive partitioning plots were generated from expression data from Taylor *et al.*,(2010) [2], which included 140 patients with prostate cancer (131 primary and 9 metastatic tumors) screened with Affymetrix human genome exon arrays, and accompanying biochemical data. Higher levels of of *HNF1B* predict positive clinical outcome (**C**), from the analysis of publicly-available gene expression profiles from 79 primary PC tumors and accompanying survival data (median 70 months) (Glinsky *et al., 2004)* [5].

SLC14A1 FLRT3

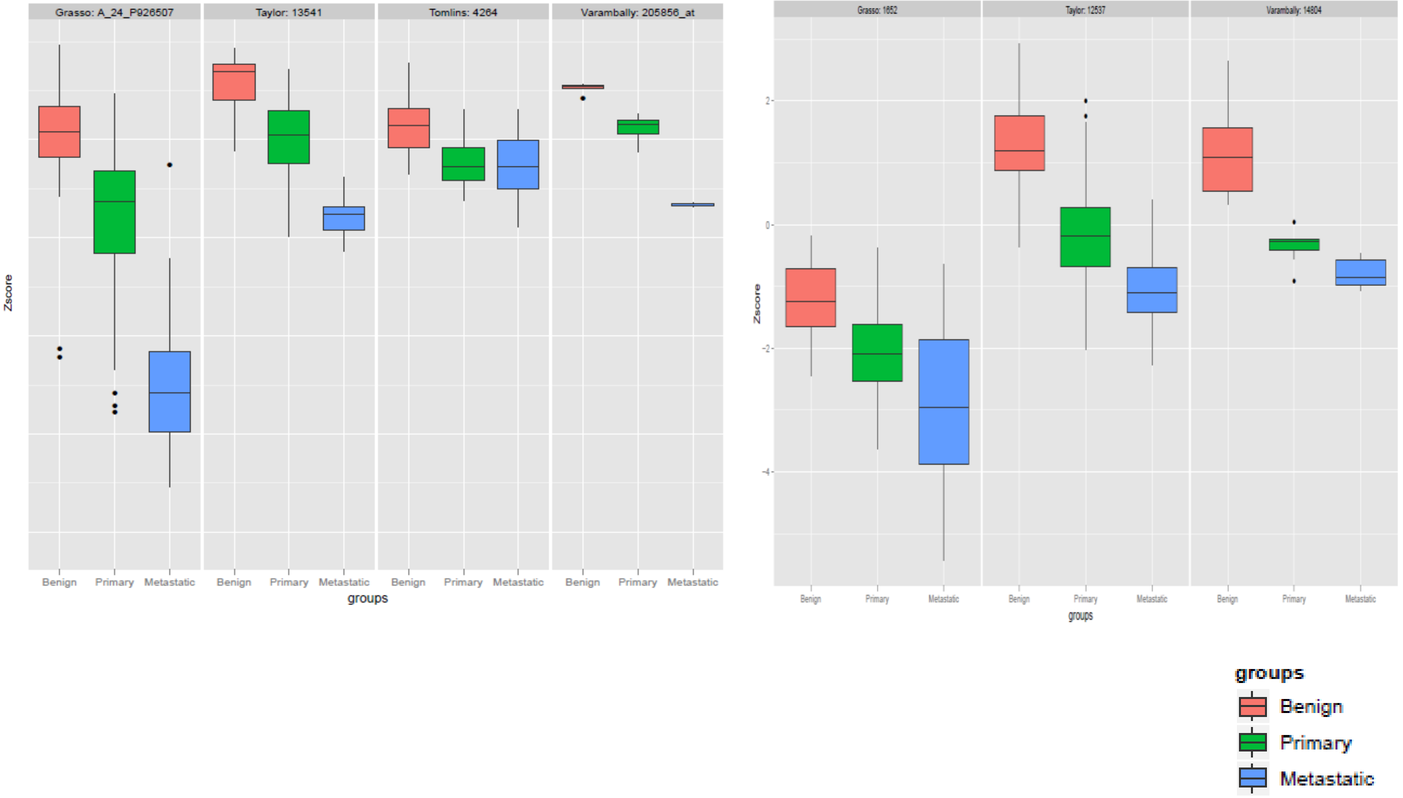

Supplementary Fig 11. Gene expression levels of *SLC14A1* and *FLRT3* decrease from benign to primary and metastatic samples in multiple clinical datasets from Grasso *et al*. (2012), Taylor *et al*. (2010), Tomlins *et al*. (2012) and Varambally *et al*. (2005) [1-4].

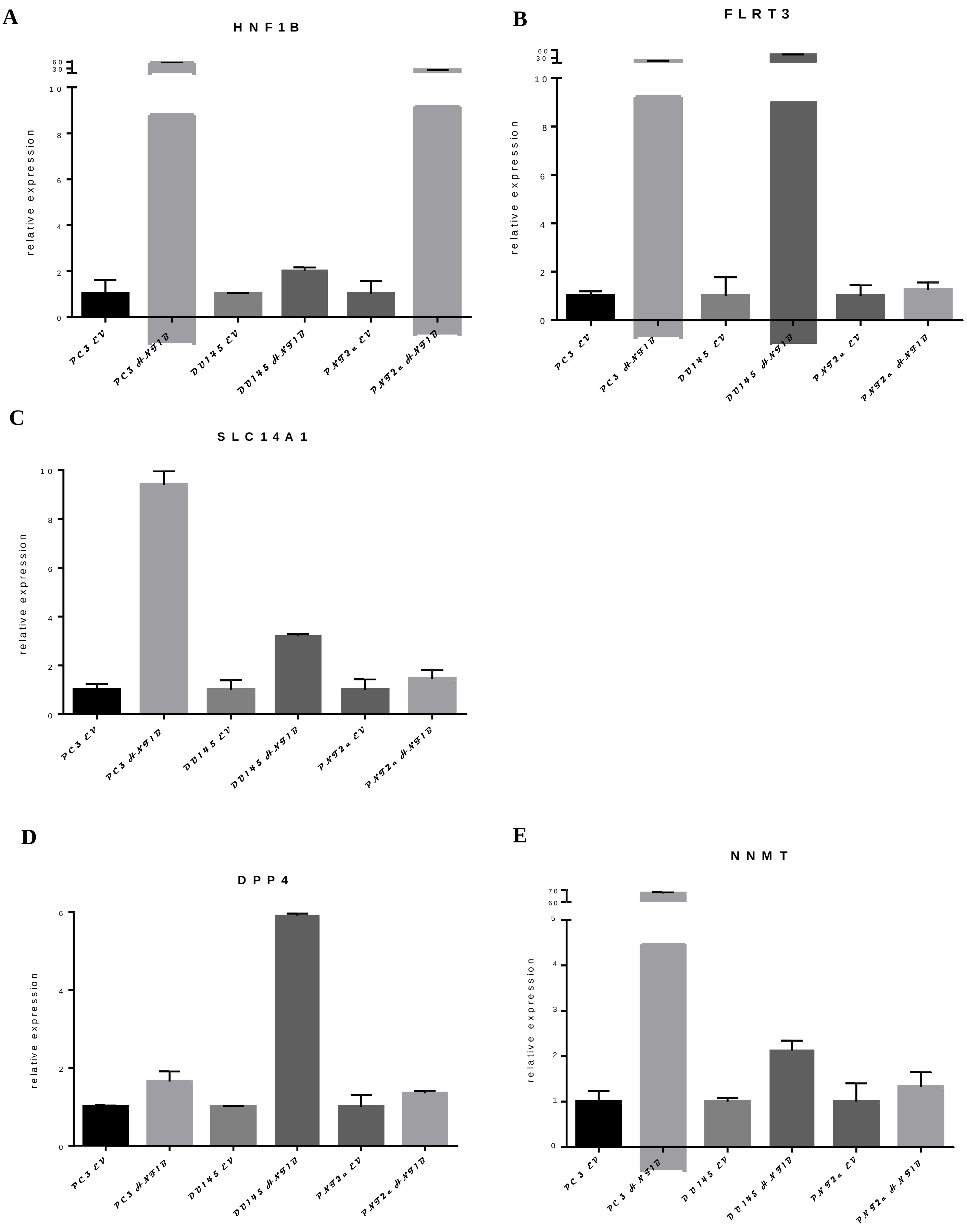
A
HNF1B
B
FLRT3
C
SLC14A1
D
DPP4
E
NNMT
relative expression
PC3 EV
PC3 HNF1B
DU145 EV
DU145 HNF1B
PNT2a EV
PNT2a HNF1B

Supplementary Fig 12. Over-expression of *HNF1B* in prostate lines was confirmed (**A**), and correlated with elevated expression levels of novel targets *FLRT3* and *SLC14A1* (**B, C**) in PC3 and DU145 models, but not in normal prostate PNT2a cells. Expression of known targets of *HNF1B, DPP4* and *NNMT* were also confirmed (**D-E**).

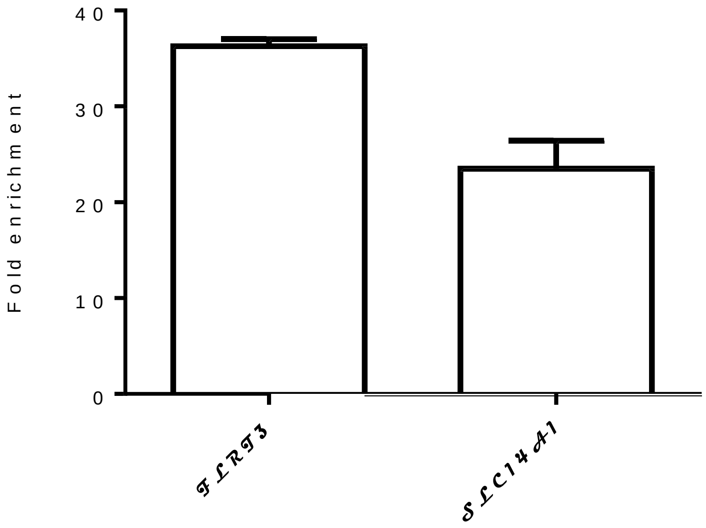

Supplementary Figure 13.
ChIP-qPCR showed enrichment of *FLRT3* and *SLC14A1* in PC3-HNF1B cells, suggesting direct interaction between transcription factor *HNF1B* and these gene targets at the chromatin level. Target enrichment was assessed by qPCR following ChIP with anti-HNF1B antibody. Data were normalized to input (no immunoprecipitation).

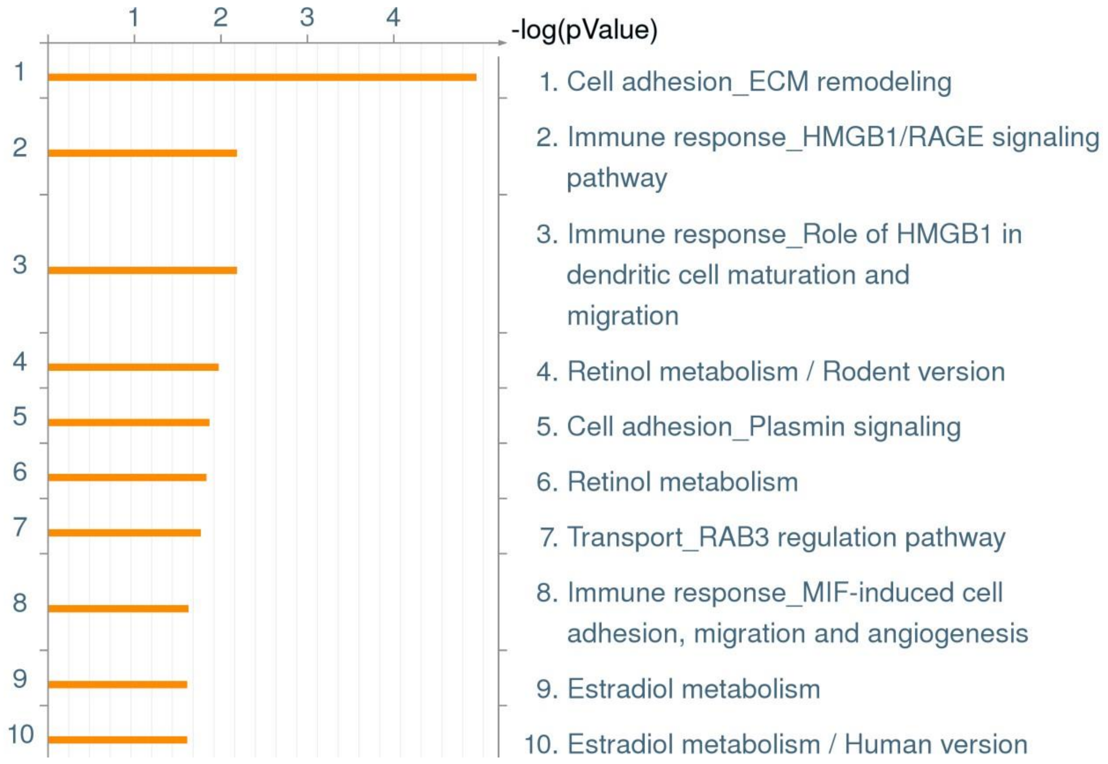

Supplementary Figure 14. The top 250 DEGs following shRNA knock-down of *HNF1B* in RMG2 ovarian cancer cells suggest that pathways involved in extra-cellular matrix remodelling and cellular adhesion is also evident in this disease model. Publicly-available data from Affymetrix HG U133 Plus 2.0 arrays were obtained from GEO (GSE37290) and analysed in GEO2R (http://w ww.ncbi .nl m.nih. gov/geo/geo2r/). Gene ontology enrichment analysis was performed in MetaCore (Thomas Reuters).

A

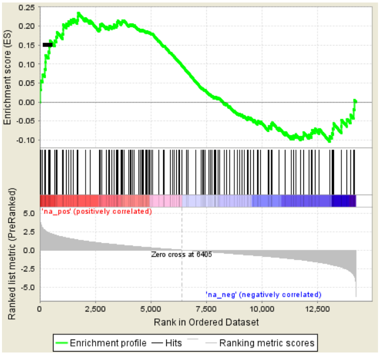

5-year survivors-deceased OvCa; NES 1.14, FWER p=0.045

B

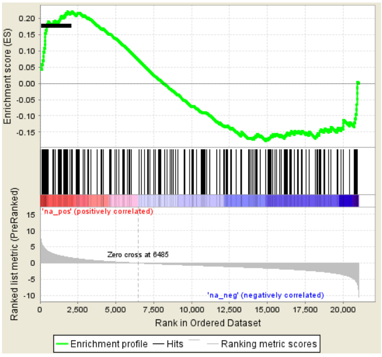

Normal-primary OvCa; NES 1.13, FWER p=0.012

Supplementary Figure 15

The 210 HNF1B-related DEGs from prostate are also significantly enriched in two ovarian cancer studies: (A) Partheen *et al.* (2006) [6], (B) Bowen *et al.* (2009) [7]. Leading edge analysis (thick black bars) identified the gene set driving enrichment in each sample comparison. For all comparisons performed, see Supplementary Table 3.

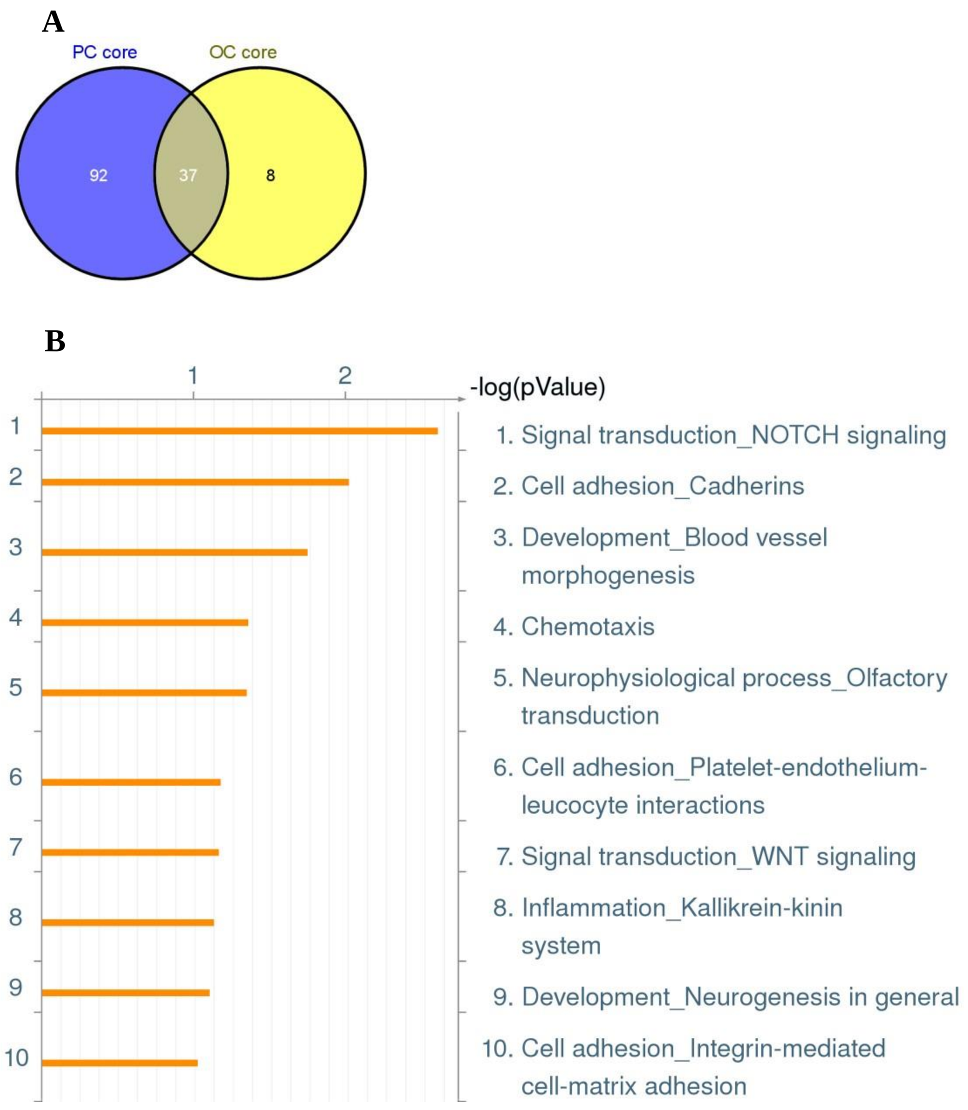

Supplementary Figure 16

A. Overlap between 129 'leading edge' genes from four prostate cancer clinical studies (PC core) [1-4] and 45 leading edge genes from two ovarian cancer clinical studies (OC core) [6, 7], identified following GSEA with the 210 HNF1B-associated DEG signature. See Supplementary Table 8 for the 37 consensus genes identified. **B.** Pathway analysis confirmed that the core processes associated with these genes relate to cadherin- and integrin-mediated adhesion and chemotaxis (MetaCore; Thomas Reuters).
