# Supplementary material for: HNF1B variants associate with promoter methylation and regulate gene networks activated in prostate and ovarian cancer
Source: Oncotarget. 2016 Oct 9;7(46):74734–46. doi: 10.18632/oncotarget.12543 (PMC5342698; doi:10.18632/oncotarget.12543)
Supplement: Supplementary file 2 [file oncotarget-07-74734-s002.doc]

*HNF1B* variants associate with promoter methylation and regulate gene networks activated in prostate and ovarian cancer

**Supplementary Material**

**Supplementary Table 1.** 210 differentially expressed genes identified from two biological replicates in PC3 HNF1B cells vs PC3 EV (‘an HNF1B-gene signature’). Initially, 4,547 DEGs were identified (false discovery rate (FDR) p<0.01). Only statistically significant genes also showing a ≥3-fold change in gene expression were considered in subsequent analyses.

| ProbeId | Symbol | RefSeq | logFC | adj.P.Val |
| --- | --- | --- | --- | --- |
| ILMN_2076600 | ITM2A | NM_004867.3 | 6.71E+00 | 4.63E-41 |
| ILMN_1744817 | UGT1A1 | NM_000463.2 | 5.60E+00 | 2.92E-33 |
| ILMN_1772894 | TMEM27 | NM_020665.3 | 5.01E+00 | 3.19E-30 |
| ILMN_1662932 | LCP1 | NM_002298.2 | 4.32E+00 | 4.18E-10 |
| ILMN_2109197 | EPB41L3 | NM_012307.2 | 4.21E+00 | 5.81E-31 |
| ILMN_2112638 | SVEP1 | NM_153366.2 | 4.04E+00 | 6.88E-25 |
| ILMN_1712082 | GCNT3 | NM_004751.1 | 3.75E+00 | 2.04E-20 |
| ILMN_1782389 | LAD1 | NM_005558.3 | 3.63E+00 | 4.10E-13 |
| ILMN_1713952 | C1orf106 | NM_018265.1 | 3.57E+00 | 3.21E-08 |
| ILMN_1796059 | ANKRD30A | XM_001131823.1 | 3.44E+00 | 3.41E-27 |
| ILMN_2212878 | ESM1 | NM_007036.3 | 3.40E+00 | 1.50E-25 |
| ILMN_1706051 | PLD5 | NM_152666.1 | 3.28E+00 | 1.63E-26 |
| ILMN_1678842 | THBS2 | NM_003247.2 | 3.19E+00 | 4.80E-25 |
| ILMN_1653028 | COL4A1 | NM_001845.4 | 3.19E+00 | 1.29E-21 |
| ILMN_2313672 | IL1RL1 | NM_003856.2 | 3.10E+00 | 1.68E-11 |
| ILMN_1696339 | ZIC2 | NM_007129.2 | 3.03E+00 | 8.47E-25 |
| ILMN_1679194 | DQ895814 | XM_001128725.1 | 2.90E+00 | 2.31E-21 |
| ILMN_1810420 | DYSF | NM_003494.2 | 2.90E+00 | 6.27E-10 |
| ILMN_1760347 | SRGN | NM_002727.2 | 2.89E+00 | 1.60E-25 |
| ILMN_1687501 | MOXD1 | NM_015529.2 | 2.76E+00 | 2.93E-23 |
| ILMN_1794492 | HOXC6 | NM_004503.3 | 2.74E+00 | 1.79E-23 |
| ILMN_1805665 | FLRT3 | NM_198391.1 | 2.66E+00 | 1.28E-15 |
| ILMN_1713124 | AKR1C3 | NM_003739.4 | 2.64E+00 | 1.86E-14 |
| ILMN_1706643 | COL6A3 | NM_057165.2 | 2.64E+00 | 1.39E-23 |
| ILMN_1686116 | THBS1 | NM_003246.2 | 2.62E+00 | 2.73E-07 |
| ILMN_1766675 | CDH6 | NM_004932.2 | 2.62E+00 | 9.00E-24 |
| ILMN_1651826 | BASP1 | NM_006317.3 | 2.58E+00 | 9.07E-07 |
| ILMN_1771652 | BAIAP2L2 | NM_025045.4 | 2.58E+00 | 2.34E-08 |
| ILMN_2131861 | SOCS2 | NM_003877.3 | 2.58E+00 | 5.92E-21 |
| ILMN_1678143 | ARHGDIB | NM_001175.4 | 2.52E+00 | 3.46E-15 |
| ILMN_1800787 | RFTN1 | NM_015150.1 | 2.50E+00 | 6.41E-19 |
| ILMN_1705750 | TGM2 | NM_004613.2 | 2.49E+00 | 2.52E-15 |
| ILMN_1709683 | RASSF2 | NM_170773.1 | 2.47E+00 | 4.18E-10 |
| ILMN_1715508 | NNMT | NM_006169.2 | 2.45E+00 | 3.47E-08 |
| ILMN_1659688 | LGALS3BP | NM_005567.2 | 2.44E+00 | 3.34E-20 |
| ILMN_1704353 | IGSF3 | NM_001542.2 | 2.44E+00 | 5.26E-20 |
| ILMN_1761733 | HLA-DMB | NM_002118.3 | 2.42E+00 | 1.45E-21 |
| ILMN_1715684 | LAMB3 | NM_000228.2 | 2.40E+00 | 2.02E-17 |
| ILMN_2157099 | CCNA1 | NM_003914.2 | 2.39E+00 | 3.87E-18 |
| ILMN_1666733 | IL8 | NM_000584.2 | 2.39E+00 | 4.65E-08 |
| ILMN_1808715 | LOC389300 | XM_943175.1 | 2.39E+00 | 3.99E-09 |

| ILMN_1712684 | FAM20C | NM_020223.2 | 2.36E+00 | 8.39E-08 |
| --- | --- | --- | --- | --- |
| ILMN_2410713 | FGFR4 | NM_213647.1 | 2.35E+00 | 2.73E-06 |
| ILMN_1781155 | LYN | NM_002350.1 | 2.33E+00 | 1.00E-21 |
| ILMN_1741430 | MAGEA10 | NM_021048.3 | 2.33E+00 | 2.85E-22 |
| ILMN_1723092 | CRB3 | NM_139161.2 | 2.32E+00 | 2.73E-07 |
| ILMN_1776516 | ITPKA | NM_002220.1 | 2.30E+00 | 9.33E-21 |
| ILMN_1696702 | NEO1 | NM_002499.1 | 2.30E+00 | 1.87E-19 |
| ILMN_1698725 | FRMD3 | NM_174938.3 | 2.30E+00 | 1.22E-22 |
| ILMN_2190084 | VAMP8 | NM_003761.2 | 2.30E+00 | 1.12E-06 |
| ILMN_1724832 | OVOL2 | NM_021220.2 | 2.30E+00 | 1.81E-09 |
| ILMN_1687757 | AKR1C1 | NM_001818.2 | 2.29E+00 | 3.25E-16 |
| ILMN_1674908 | HOXB5 | NM_002147.3 | 2.28E+00 | 6.42E-21 |
| ILMN_1748303 | MUC5AC | XM_001134429.1 | 2.28E+00 | 2.12E-21 |
| ILMN_2302757 | FCGBP | NM_003890.1 | 2.28E+00 | 6.96E-18 |
| ILMN_1782939 | DQ894588 | NM_000477.3 | 2.25E+00 | 4.21E-21 |
| ILMN_2083946 | TGFA | NM_003236.1 | 2.24E+00 | 4.00E-19 |
| ILMN_1854469 | AK130049 |  | 2.23E+00 | 1.65E-20 |
| ILMN_1778401 | HLA-B | NM_005514.5 | 2.21E+00 | 2.35E-17 |
| ILMN_1658498 | SLC44A3 | NM_152369.2 | 2.15E+00 | 8.34E-18 |
| ILMN_1792978 | HAS2 | NM_005328.1 | 2.13E+00 | 8.98E-21 |
| ILMN_1781761 | ENPP4 | NM_014936.3 | 2.11E+00 | 9.06E-20 |
| ILMN_2367258 | SMOX | NM_175840.1 | 2.11E+00 | 2.40E-05 |
| ILMN_2285404 | DMKN | NM_033317.2 | 2.09E+00 | 2.51E-06 |
| ILMN_1706483 | C1orf116 | NM_023938.5 | 2.08E+00 | 6.18E-09 |
| ILMN_1779234 | CXCL6 | NM_002993.2 | 2.07E+00 | 2.37E-12 |
| ILMN_2412336 | AKR1C2 | NM_001354.4 | 2.07E+00 | 2.30E-09 |
| ILMN_2199768 | SLITRK4 | NM_173078.2 | 2.06E+00 | 2.04E-20 |
| ILMN_1667199 | SQRDL | NM_021199.2 | 2.06E+00 | 5.64E-07 |
| ILMN_1808379 | PLCD4 | NM_032726.2 | 2.05E+00 | 5.41E-21 |
| ILMN_2069632 | GTSF1 | NM_144594.1 | 2.04E+00 | 3.91E-20 |
| ILMN_1695404 | LY6E | NM_002346.1 | 2.04E+00 | 1.63E-19 |
| ILMN_1744937 | PTPRM | NM_002845.2 | 2.03E+00 | 9.38E-17 |
| ILMN_1672148 | EU176471 | NM_020299.3 | 2.03E+00 | 5.30E-23 |
| ILMN_1733998 | DHRS9 | NM_005771.3 | 2.03E+00 | 4.19E-08 |
| ILMN_1678049 | FGB | NM_005141.2 | 2.02E+00 | 2.12E-21 |
| ILMN_1804351 | FZD7 | NM_003507.1 | 2.00E+00 | 2.29E-17 |
| ILMN_1730645 | TMEFF2 | NM_016192.2 | 1.99E+00 | 1.79E-23 |
| ILMN_2139396 | IGDCC4 | NM_020962.1 | 1.97E+00 | 8.28E-20 |
| ILMN_1663866 | TGFBI | NM_000358.1 | 1.95E+00 | 2.88E-18 |
| ILMN_1703886 | SLC16A2 | NM_006517.2 | 1.95E+00 | 1.34E-05 |
| ILMN_2314169 | PTHLH | NM_198965.1 | 1.94E+00 | 2.38E-12 |
| ILMN_1718285 | HOXC8 | NM_022658.3 | 1.94E+00 | 9.52E-18 |
| ILMN_1854469 |  |  | 1.93E+00 | 5.02E-04 |
| ILMN_1707339 | BTG3 | NM_006806.3 | 1.92E+00 | 2.08E-20 |
| ILMN_1684886 | VCX | NM_013452.2 | 1.92E+00 | 3.92E-09 |
| ILMN_1763837 | ANPEP | NM_001150.1 | 1.91E+00 | 1.63E-17 |

| ILMN_1782439 | CNN3 | NM_001839.2 | 1.90E+00 | 2.00E-18 |
| --- | --- | --- | --- | --- |
| ILMN_2166716 | VCX-C | NM_001001888.1 | 1.89E+00 | 1.69E-07 |
| ILMN_1791531 | FA2H | NM_024306.2 | 1.87E+00 | 1.61E-06 |
| ILMN_1672148 | AKR1B10 | NM_020299.3 | 1.86E+00 | 5.84E-07 |
| ILMN_2188264 | CYR61 | NM_001554.3 | 1.85E+00 | 1.17E-16 |
| ILMN_1675684 | APOBEC3C | NM_014508.2 | 1.85E+00 | 2.51E-06 |
| ILMN_1655614 | DSP | NM_001008844.1 | 1.83E+00 | 6.31E-17 |
| ILMN_1673769 | KCNG1 | NM_002237.3 | 1.83E+00 | 1.14E-05 |
| ILMN_1718766 | MT1F | NM_005949.2 | 1.83E+00 | 4.37E-04 |
| ILMN_2407824 | ATP1B1 | NM_001001787.1 | 1.83E+00 | 9.74E-18 |
| ILMN_1789502 | GPC4 | NM_001448.2 | 1.82E+00 | 2.77E-19 |
| ILMN_2413816 | GRB14 | NM_004490.2 | 1.82E+00 | 7.44E-18 |
| ILMN_1701933 | SNCA | NM_007308.1 | 1.81E+00 | 3.23E-18 |
| ILMN_1679194 | UGT2B7 | XM_001128725.1 | 1.81E+00 | 2.30E-09 |
| ILMN_2406035 | LAMA3 | NM_198129.1 | 1.81E+00 | 2.20E-10 |
| ILMN_1752520 | SLFN11 | NM_152270.2 | 1.80E+00 | 3.42E-17 |
| ILMN_2352090 | GPRC5C | NM_018653.3 | 1.79E+00 | 1.87E-03 |
| ILMN_2160210 | EPCAM | NM_002354.1 | 1.79E+00 | 3.09E-19 |
| ILMN_1784661 | KIAA1412 | NM_013390.1 | 1.78E+00 | 8.99E-18 |
| ILMN_1667711 | DQ895783 | NM_007069.2 | 1.78E+00 | 2.03E-19 |
| ILMN_2081087 | HSPA12A | NM_025015.2 | 1.77E+00 | 9.33E-21 |
| ILMN_1706498 | DSE | NM_013352.2 | 1.77E+00 | 1.27E-17 |
| ILMN_1708093 | ARHGEF5 | NM_005435.3 | 1.76E+00 | 1.09E-04 |
| ILMN_2175912 | ITGB2 | NM_000211.2 | 1.75E+00 | 1.75E-08 |
| ILMN_1703572 | PCDH20 | NM_022843.2 | 1.74E+00 | 2.29E-20 |
| ILMN_2366642 | VCX3A | NM_016379.2 | 1.74E+00 | 4.32E-08 |
| ILMN_1675797 | EPDR1 | NM_017549.3 | 1.74E+00 | 4.05E-18 |
| ILMN_1705403 | CYP2S1 | NM_030622.6 | 1.73E+00 | 6.30E-04 |
| ILMN_2214473 | ARHGEF5L | NM_001003702.1 | 1.73E+00 | 8.42E-18 |
| ILMN_2214473 | FLJ43692 | NM_001003702.1 | 1.70E+00 | 2.15E-07 |
| ILMN_1677814 | ABCC3 | NM_003786.2 | 1.69E+00 | 1.56E-18 |
| ILMN_1666845 | KRT17 | NM_000422.1 | 1.69E+00 | 2.73E-06 |
| ILMN_1685714 | INHBB | NM_002193.1 | 1.69E+00 | 3.10E-20 |
| ILMN_1707975 | SERPIND1 | NM_000185.3 | 1.69E+00 | 3.66E-20 |
| ILMN_1672662 | SLC20A1 | NM_005415.3 | 1.67E+00 | 1.40E-14 |
| ILMN_1663919 | TFF2 | NM_005423.3 | 1.66E+00 | 1.96E-17 |
| ILMN_1769245 | GLIPR1 | NM_006851.2 | 1.64E+00 | 2.63E-17 |
| ILMN_1663080 | LFNG | NM_001040167.1 | 1.62E+00 | 7.95E-08 |
| ILMN_1780132 | PELI2 | NM_021255.2 | 1.62E+00 | 8.41E-16 |
| ILMN_1674415 | LOC650463 | XM_939552.1 | 1.62E+00 | 1.82E-07 |
| ILMN_1769575 | JAM3 | NM_032801.3 | 1.60E+00 | 3.07E-17 |
| ILMN_1656057 | PLAU | NM_002658.2 | 1.60E+00 | 1.11E-12 |
| ILMN_1814327 | AGTR1 | NM_000685.4 | 1.60E+00 | 5.26E-20 |
| ILMN_1653856 | UBASH3B | NM_032873.3 | 1.60E+00 | 8.56E-15 |
| ILMN_1873278 | FAM155A | XM_001133620.1 | 1.59E+00 | 3.69E-19 |
| ILMN_1799198 | OTUB2 | NM_023112.2 | 1.59E+00 | 6.81E-05 |

| ILMN_1801216 | S100P | NM_005980.2 | 1.59E+00 | 4.71E-04 |
| --- | --- | --- | --- | --- |
| ILMN_1800697 | LDB2 | NM_001290.2 | 1.59E+00 | 8.07E-08 |
| ILMN_1743130 | PTGFRN | NM_020440.2 | 1.58E+00 | 2.35E-17 |
| ILMN_1664660 | GAGE1 | NM_001098409.1 | 1.57E+00 | 5.09E-17 |
| ILMN_1705442 | CMTM3 | NM_144601.2 | 1.57E+00 | 1.88E-16 |
| ILMN_1699772 | RRAGD | NM_021244.3 | 1.57E+00 | 9.82E-16 |
| ILMN_1691341 | IL7R | XM_937367.1 | 1.56E+00 | 1.50E-12 |
| ILMN_2251766 | IL1R2 | NM_173343.1 | 1.56E+00 | 1.75E-07 |
| ILMN_1667114 | LOC388524 | NR_003662.1 | 1.56E+00 | 2.64E-08 |
| ILMN_1760414 | AADAC | NM_001086.2 | 1.54E+00 | 1.15E-16 |
| ILMN_1724994 | COL4A2 | NM_001846.2 | 1.54E+00 | 6.83E-13 |
| ILMN_1668194 | LMTK3 | XM_936372.2 | 1.53E+00 | 2.32E-04 |
| ILMN_1772466 | SH2D3A | NM_005490.1 | 1.53E+00 | 6.35E-06 |
| ILMN_1656560 | DKFZP564O0823 | NM_015393.2 | 1.52E+00 | 2.44E-16 |
| ILMN_1674985 | TMEM51 | NM_018022.1 | 1.52E+00 | 3.98E-17 |
| ILMN_1656910 | TRIM6 | NM_058166.3 | 1.52E+00 | 5.99E-12 |
| ILMN_1805561 | SLC14A1 | NM_015865.2 | 1.51E+00 | 8.44E-14 |
| ILMN_1672589 | SEMA4B | NM_198925.1 | 1.51E+00 | 6.26E-15 |
| ILMN_1852151 | BX110351 |  | 1.50E+00 | 3.55E-16 |
| ILMN_1730639 | SLC22A15 | NM_018420.1 | 1.50E+00 | 5.03E-15 |
| ILMN_1667068 | ZC3HAV1 | NM_020119.3 | -2.69E-01 | 9.95E-03 |
| ILMN_2089656 | C1orf107 | NM_014388.5 | -2.93E-01 | 9.94E-03 |
| ILMN_1774083 | TRIAP1 | NM_016399.2 | -2.97E-01 | 9.95E-03 |
| ILMN_1655497 | EIF4B | NM_001417.4 | -3.10E-01 | 9.96E-03 |
| ILMN_1764127 | NPHP1 | NM_207181.1 | -3.16E-01 | 9.97E-03 |
| ILMN_1770031 | ABHD10 | NM_018394.1 | -3.58E-01 | 9.94E-03 |
| ILMN_1660871 | NEK6 | NM_014397.3 | -3.64E-01 | 9.94E-03 |
| ILMN_1725594 | C10orf97 | NM_024948.2 | -1.50E+00 | 2.10E-15 |
| ILMN_1704294 | CDH3 | NM_001793.3 | -1.50E+00 | 6.05E-12 |
| ILMN_1664798 | GRHPR | NM_012203.1 | -1.51E+00 | 7.72E-18 |
| ILMN_1808661 | TOMM5 | NM_001001790.2 | -1.54E+00 | 1.46E-16 |
| ILMN_1786197 | NR2F1 | NM_005654.4 | -1.54E+00 | 5.67E-06 |
| ILMN_1777660 | RNF144 | NM_014746.2 | -1.56E+00 | 1.20E-05 |
| ILMN_1682273 | SHISA3 | NM_001080505.1 | -1.58E+00 | 2.20E-07 |
| ILMN_1781010 | ARHGEF3 | NM_019555.1 | -1.60E+00 | 6.99E-16 |
| ILMN_1807894 | SLC7A8 | NM_182728.1 | -1.60E+00 | 6.05E-19 |
| ILMN_1723969 | PLCB1 | NM_015192.2 | -1.61E+00 | 9.00E-17 |
| ILMN_1787932 | GPR110 | NM_025048.2 | -1.61E+00 | 1.06E-09 |
| ILMN_2148913 | TMEM45A | NM_018004.1 | -1.62E+00 | 3.47E-12 |
| ILMN_1708936 | EXOSC3 | NM_016042.2 | -1.62E+00 | 8.42E-18 |
| ILMN_1678934 | POLR1E | NM_022490.1 | -1.62E+00 | 7.92E-15 |
| ILMN_1724789 | CD59 | NM_000611.4 | -1.66E+00 | 4.75E-07 |
| ILMN_2380938 | SYT7 | NM_004200.2 | -1.66E+00 | 1.72E-15 |
| ILMN_2138745 | C14orf37 | NM_001001872.2 | -1.67E+00 | 1.17E-16 |
| ILMN_1725139 | CA9 | NM_001216.1 | -1.69E+00 | 5.43E-12 |
| ILMN_1677273 | TH | NM_199293.2 | -1.70E+00 | 5.15E-05 |

| ILMN_1753005 | RELN | NM_005045.2 | -1.71E+00 | 6.75E-20 |
| --- | --- | --- | --- | --- |
| ILMN_1788250 | LDOC1 | NM_012317.2 | -1.71E+00 | 4.93E-18 |
| ILMN_1814221 | NPTX1 | NM_002522.2 | -1.73E+00 | 2.25E-18 |
| ILMN_1760247 | CD70 | NM_001252.3 | -1.73E+00 | 1.19E-05 |
| ILMN_1685699 | PRSS3 | NM_002771.2 | -1.75E+00 | 9.89E-19 |
| ILMN_1721247 | KRT75 | NM_004693.2 | -1.75E+00 | 1.33E-18 |
| ILMN_2169383 | REG4 | NM_032044.2 | -1.79E+00 | 1.51E-05 |
| ILMN_1800540 | CD55 | NM_000574.2 | -1.80E+00 | 2.97E-03 |
| ILMN_1720048 | CCL2 | NM_002982.3 | -1.82E+00 | 2.59E-08 |
| ILMN_1744381 | SERPINE1 | NM_000602.1 | -1.84E+00 | 1.60E-06 |
| ILMN_1687384 | IFI6 | NM_022873.2 | -1.85E+00 | 1.51E-03 |
| ILMN_1777660 | RNF144A | NM_014746.2 | -1.90E+00 | 3.88E-20 |
| ILMN_1713266 | FAM46C | NM_017709.3 | -1.94E+00 | 2.16E-19 |
| ILMN_2331231 | TNFRSF6B | NM_032945.2 | -1.95E+00 | 3.44E-07 |
| ILMN_1768646 | AK056814 | NM_182510.1 | -1.95E+00 | 1.01E-19 |
| ILMN_1656415 | CDKN2C | NM_078626.2 | -1.97E+00 | 2.37E-19 |
| ILMN_1676563 | HTRA1 | NM_002775.3 | -2.07E+00 | 3.88E-07 |
| ILMN_1726597 | FAM65B | NM_015864.2 | -2.10E+00 | 8.42E-21 |
| ILMN_1758067 | RGS4 | NM_005613.3 | -2.10E+00 | 3.17E-06 |
| ILMN_1765860 | DOCK11 | NM_144658.3 | -2.12E+00 | 2.27E-22 |
| ILMN_1683263 | TSPAN8 | NM_004616.2 | -2.15E+00 | 9.15E-05 |
| ILMN_1688780 | S100A4 | NM_019554.2 | -2.17E+00 | 5.67E-06 |
| ILMN_1711988 | KCNK12 | NM_022055.1 | -2.38E+00 | 5.69E-25 |
| ILMN_1727466 | KCNMB4 | NM_014505.4 | -2.44E+00 | 1.35E-21 |
| ILMN_1738742 | PLAT | NM_000930.2 | -2.51E+00 | 5.50E-09 |
| ILMN_1740426 | RASD1 | NM_016084.3 | -2.53E+00 | 9.15E-25 |
| ILMN_1666222 | PHACTR3 | NM_183246.1 | -2.71E+00 | 1.09E-07 |
| ILMN_1674063 | OAS2 | NM_016817.2 | -2.79E+00 | 1.46E-03 |
| ILMN_1805376 | KCNJ6 | NM_002240.2 | -3.08E+00 | 3.16E-26 |
| ILMN_2180232 | URP | NM_198152.2 | -3.19E+00 | 1.34E-26 |
| ILMN_1662358 | MX1 | NM_002462.2 | -3.36E+00 | 1.98E-04 |
| ILMN_1725417 | NELL2 | NM_006159.1 | -3.49E+00 | 7.96E-31 |
| ILMN_2058782 | IFI27 | NM_005532.3 | -3.83E+00 | 4.22E-04 |

**Supplementary Table 2.** Data sets included in the gene set enrichment analysis of 210

*HNF1B* gene signature in publicly-available prostate and ovarian cancer clinical studies

| **Study** | **Sample group comparison** | **Enrichment**  **(top/bottom)*** | **Normalized**  **enrichment scores (NES)** | **FWER**  **p-value** |
| --- | --- | --- | --- | --- |
| Glinsky *et al*., 2004 | Benign-primary PC | Bottom | -1.02 | 0.445 |
| Varambally *et al*., 2005 | Benign-primary PC | Top | 1.36 | <0.001 |
|  | Benign-metastatic PC | Top | 1.60 | <0.001 |
|  | Primary-metastatic PC | Top | 1.47 | <0.001 |
| Tomlins *et al*., 2007 | Normal-primary PC | Top | 1.25 | 0.015 |
| Taylor *et al*., 2010 | Normal-primary PC | top | 1.79 | <0.001 |
|  | Normal-metastatic PC | Top | 1.78 | <0.001 |
|  | Primary-metastatic PC | Top | 1.47 | <0.001 |
| Grasso *et al*., 2012 | Benign-primary PC | Top | 1.75 | <0.001 |
|  | Benign-metastatic PC | Top | 1.94 | <0.001 |
|  | Primary-metastatic PC | Top | 1.80 | <0.001 |
| Bowen *et al.,* 2009 | Normal-primary OC | Top | 1.13 | 0.012 |
| Partheen *et al.*, 2006 | 5-year survivors-deceased OC | Top | 1.14 | 0.045 |

* Study expression profiles for each sample group comparison were ranked in ascending order according to t-statistic, so over- or under-expressed targets could be distinguished using family-wise error rate (FWER) p-value (See [www.broadinstitute.org/gsea](http://www.broadinstitute.org/gsea) and (Benjamini & Hochberg, 1995) [8]). Here, the ‘top’ of the distribution corresponds to the first phenotype (benign); the bottom of the distribution corresponds to the second phenotype (disease state).

**Supplementary Table 3**. 129 key, functionally relevant genes were identified by ‘leading edge’ analysis of the *HNF1B* gene signature across four clinical prostate cancer datasets [1-4] using GSEA ([www.broadinstitute.org/gsea](http://www.broadinstitute.org/gsea)).

| Symbol | Entrez Gene Name |
| --- | --- |
| ABCC3 | ATP-binding cassette, sub-family C (CFTR/MRP), member 3 |
| ABHD10 | abhydrolase domain containing 10 |
| AGTR1 | angiotensin II receptor, type 1 |
| AKR1C1 | aldo-keto reductase family 1, member C1 |
| AKR1C2 | aldo-keto reductase family 1, member C2 |
| AKR1C3 | aldo-keto reductase family 1, member C3 |
| ANPEP | alanyl (membrane) aminopeptidase |
| APOBEC3C | apolipoprotein B mRNA editing enzyme, catalytic polypeptide-like 3C |
| ARHGDIB | Rho GDP dissociation inhibitor (GDI) beta |
| ARHGEF3 | Rho guanine nucleotide exchange factor (GEF) 3 |
| ATP1B1 | ATPase, Na+/K+ transporting, beta 1 polypeptide |
| BASP1 | brain abundant, membrane attached signal protein 1 |
| BTG3 | BTG family, member 3 |
| C14orf37 | chromosome 14 open reading frame 37 |
| C1orf106 | chromosome 1 open reading frame 106 |
| C1orf116 | chromosome 1 open reading frame 116 |
| CA9 | carbonic anhydrase IX |
| CCL2 | chemokine (C-C motif) ligand 2 |
| CD55 | CD55 molecule, decay accelerating factor for complement (Cromer blood group) |
| CD59 | CD59 molecule, complement regulatory protein |
| CDH3 | cadherin 3, type 1, P-cadherin (placental) |
| CDH6 | cadherin 6, type 2, K-cadherin (fetal kidney) |
| CDKN2C | cyclin-dependent kinase inhibitor 2C (p18, inhibits CDK4) |
| CNN3 | calponin 3, acidic |
| COL4A1 | collagen, type IV, alpha 1 |
| COL4A2 | collagen, type IV, alpha 2 |
| COL6A3 | collagen, type VI, alpha 3 |
| CXCL6 | chemokine (C-X-C motif) ligand 6 |
| CYR61 | cysteine-rich, angiogenic inducer, 61 |
| DHRS9 | dehydrogenase/reductase (SDR family) member 9 |
| DMKN | dermokine |
| DOCK11 | dedicator of cytokinesis 11 |
| DSE | dermatan sulfate epimerase |
| DSP | desmoplakin |
| DYSF | dysferlin, limb girdle muscular dystrophy 2B (autosomal recessive) |
| EIF4B | eukaryotic translation initiation factor 4B |
| ENPP4 | ectonucleotide pyrophosphatase/phosphodiesterase 4 (putative) |

| EPB41L3 | erythrocyte membrane protein band 4.1-like 3 |
| --- | --- |
| EPDR1 | ependymin related protein 1 (zebrafish) |
| FA2H | fatty acid 2-hydroxylase |
| FAM155A | family with sequence similarity 155, member A |
| FAM20C | family with sequence similarity 20, member C |
| FAM46C | family with sequence similarity 46, member C |
| FAM65B | family with sequence similarity 65, member B |
| FCGBP | Fc fragment of IgG binding protein |
| FLRT3 | fibronectin leucine rich transmembrane protein 3 |
| FZD7 | frizzled family receptor 7 |
| GLIPR1 | GLI pathogenesis-related 1 |
| GPC4 | glypican 4 |
| GRHPR | glyoxylate reductase/hydroxypyruvate reductase |
| HAS2 | hyaluronan synthase 2 |
| HLA-B | major histocompatibility complex, class I, B |
| HLA-DMB | major histocompatibility complex, class II, DM beta |
| HOXB5 | homeobox B5 |
| HSPA12A | heat shock 70kDa protein 12A |
| HTRA1 | HtrA serine peptidase 1 |
| IFI27 | interferon, alpha-inducible protein 27 |
| IFI6 | interferon, alpha-inducible protein 6 |
| IGDCC4 | immunoglobulin superfamily, DCC subclass, member 4 |
| IGSF3 | immunoglobulin superfamily, member 3 |
| IL1R2 | interleukin 1 receptor, type II |
| IL1RL1 | interleukin 1 receptor-like 1 |
| IL7R | interleukin 7 receptor |
| ITM2A | integral membrane protein 2A |
| ITPKA | inositol-trisphosphate 3-kinase A |
| JAM3 | junctional adhesion molecule 3 |
| KCNG1 | potassium voltage-gated channel, subfamily G, member 1 |
| KCNJ6 | potassium inwardly-rectifying channel, subfamily J, member 6 |
| KCNMB4 | potassium large conductance calcium-activated channel, subfamily M, beta member 4 |
| KRT17 | keratin 17 |
| KRT75 | keratin 75 |
| LAMA3 | laminin, alpha 3 |
| LAMB3 | laminin, beta 3 |
| LCP1 | lymphocyte cytosolic protein 1 (L-plastin) |
| LDB2 | LIM domain binding 2 |
| LDOC1 | leucine zipper, down-regulated in cancer 1 |
| LGALS3BP | lectin, galactoside-binding, soluble, 3 binding protein |
| LMTK3 | lemur tyrosine kinase 3 |
| LY6E | lymphocyte antigen 6 complex, locus E |
| MOXD1 | monooxygenase, DBH-like 1 |
| MT1F | metallothionein 1F |
| MX1 | myxovirus (influenza virus) resistance 1, interferon-inducible protein p78 (mouse) |
| NEK6 | NIMA-related kinase 6 |

| NELL2 | NEL-like 2 (chicken) |
| --- | --- |
| NEO1 | neogenin 1 |
| NPHP1 | nephronophthisis 1 (juvenile) |
| NPTX1 | neuronal pentraxin I |
| NR2F1 | nuclear receptor subfamily 2, group F, member 1 |
| OAS2 | 2'-5'-oligoadenylate synthetase 2, 69/71kDa |
| PCDH20 | protocadherin 20 |
| PELI2 | pellino E3 ubiquitin protein ligase family member 2 |
| PLAT | plasminogen activator, tissue |
| PLAU | plasminogen activator, urokinase |
| PLCB1 | phospholipase C, beta 1 (phosphoinositide-specific) |
| PLCD4 | phospholipase C, delta 4 |
| PLD5 | phospholipase D family, member 5 |
| POLR1E | polymerase (RNA) I polypeptide E, 53kDa |
| PTGFRN | prostaglandin F2 receptor negative regulator |
| PTPRM | protein tyrosine phosphatase, receptor type, M |
| RASD1 | RAS, dexamethasone-induced 1 |
| RASSF2 | Ras association (RalGDS/AF-6) domain family member 2 |
| REG4 | regenerating islet-derived family, member 4 |
| RFTN1 | raftlin, lipid raft linker 1 |
| RGS4 | regulator of G-protein signaling 4 |
| S100A4 | S100 calcium binding protein A4 |
| SEMA4B | sema domain, immunoglobulin domain (Ig), transmembrane domain (TM) and short  cytoplasmic domain, (semaphorin) 4B |
| SH2D3A | SH2 domain containing 3A |
| SLC14A1 | solute carrier family 14 (urea transporter), member 1 (Kidd blood group) |
| SLC16A2 | solute carrier family 16, member 2 (thyroid hormone transporter) |
| SLC22A15 | solute carrier family 22, member 15 |
| SLC44A3 | solute carrier family 44, member 3 |
| SLC7A8 | solute carrier family 7 (amino acid transporter light chain, L system), member 8 |
| SLFN11 | schlafen family member 11 |
| SLITRK4 | SLIT and NTRK-like family, member 4 |
| SNCA | synuclein, alpha (non A4 component of amyloid precursor) |
| SOCS2 | suppressor of cytokine signaling 2 |
| SQRDL | sulfide quinone reductase-like (yeast) |
| SRGN | serglycin |
| TGFA | transforming growth factor, alpha |
| TGFBI | transforming growth factor, beta-induced, 68kDa |
| THBS1 | thrombospondin 1 |
| TMEM27 | transmembrane protein 27 |
| TMEM45A | transmembrane protein 45A |
| TMEM51 | transmembrane protein 51 |
| TNFRSF6B | tumor necrosis factor receptor superfamily, member 6b, decoy |
| TRIAP1 | TP53 regulated inhibitor of apoptosis 1 |
| TRIM6 | tripartite motif containing 6 |
| TSPAN8 | tetraspanin 8 |

VAMP8 vesicle-associated membrane protein 8

**Supplementary Table 4.** GSEA of *HNF1B* gene signature in Partheen et al. (2006) OvCa dataset [6]. *SLC14A1* is marginally significant and highlighted in bold (truncated at 100). The rank metric score is a measure of the position of the gene (*HNF1B* gene signature) in the ranked gene list (clinical expression dataset). The enrichment score reflects the degree to which a gene set is overrepresented at the top or bottom of a ranked list of genes.

| PROBE | RANK METRIC SCORE | RUNNING ES | CORE ENRICHMENT |
| --- | --- | --- | --- |
| CDH3 | 4.644183159 | 0.03440123 | Yes |
| SH2D3A | 3.423933029 | 0.056671757 | Yes |
| SOCS2 | 2.936975718 | 0.07309105 | Yes |
| S100P | 2.658154249 | 0.08592945 | Yes |
| BAIAP2L2 | 2.601016045 | 0.10422841 | Yes |
| NEK6 | 2.582150698 | 0.12316636 | Yes |
| KCNK12 | 2.354278564 | 0.13223599 | Yes |
| ITM2A | 2.295039177 | 0.14568613 | Yes |
| FA2H | 2.27689743 | 0.1616964 | Yes |
| SMOX | 2.048242807 | 0.1617306 | Yes |
| SNCA | 1.987776756 | 0.17145751 | Yes |
| PHACTR3 | 1.950957775 | 0.18374643 | Yes |
| PLAT | 1.759124875 | 0.17905824 | Yes |
| CA9 | 1.746774673 | 0.19095118 | Yes |
| APOBEC3C | 1.745029688 | 0.20396629 | Yes |
| HTRA1 | 1.648367882 | 0.20639418 | Yes |
| CYR61 | 1.6014781 | 0.21208899 | Yes |
| NELL2 | 1.54125905 | 0.21577127 | Yes |
| NPTX1 | 1.48114574 | 0.21921562 | Yes |
| SLC7A8 | 1.38239634 | 0.21610136 | Yes |
| ANPEP | 1.368070245 | 0.22458677 | Yes |
| TGFA | 1.363510013 | 0.23431513 | Yes |
| KRT17 | 1.220762372 | 0.22055213 | No |
| **SLC14A1** | **1.137140989** | **0.21396679** | **No** |
| PCDH20 | 0.985651016 | 0.19297737 | No |
| C1orf107 | 0.968404114 | 0.19718853 | No |
| INHBB | 0.968165517 | 0.20430695 | No |
| FGB | 0.939397454 | 0.20610106 | No |
| NR2F1 | 0.867946327 | 0.19799368 | No |
| LAD1 | 0.836478651 | 0.1968165 | No |
| NNMT | 0.824350417 | 0.20016025 | No |
| FGFR4 | 0.788387001 | 0.19869338 | No |
| CDKN2C | 0.767320096 | 0.19884232 | No |
| SERPIND1 | 0.766605139 | 0.20437828 | No |
| DOCK11 | 0.734304667 | 0.20193821 | No |

| PLCB1 | 0.723803222 | 0.20523748 | No |
| --- | --- | --- | --- |
| JAM3 | 0.721637964 | 0.21001051 | No |
| TSPAN8 | 0.714207709 | 0.21366353 | No |
| CCL2 | 0.668273807 | 0.20789021 | No |
| THBS1 | 0.660217524 | 0.21113837 | No |
| SLITRK4 | 0.654016793 | 0.21462382 | No |
| C10orf97 | 0.634625554 | 0.21448721 | No |
| SVEP1 | 0.577409685 | 0.2029949 | No |
| EXOSC3 | 0.56545943 | 0.20375864 | No |
| HOXB5 | 0.550719142 | 0.20455377 | No |
| AKR1C1 | 0.533076704 | 0.20408134 | No |
| HOXC6 | 0.509748161 | 0.20144732 | No |
| SQRDL | 0.459299028 | 0.19233309 | No |
| ITGB2 | 0.439326257 | 0.18973859 | No |
| EPB41L3 | 0.423674345 | 0.1883748 | No |
| ARHGEF3 | 0.413964123 | 0.18963438 | No |
| UGT2B7 | 0.399315774 | 0.18830079 | No |
| GLIPR1 | 0.381995916 | 0.18556017 | No |
| ENPP4 | 0.368608475 | 0.18463486 | No |
| VAMP8 | 0.362636358 | 0.1857224 | No |
| ZIC2 | 0.347321212 | 0.18357317 | No |
| ANKRD30A | 0.337889284 | 0.18291417 | No |
| TGM2 | 0.322476983 | 0.1806496 | No |
| LYN | 0.306531936 | 0.17904593 | No |
| EIF4B | 0.239444152 | 0.16168444 | No |
| RRAGD | 0.190574139 | 0.14722028 | No |
| RASSF2 | 0.183523953 | 0.14675178 | No |
| AGTR1 | 0.182779357 | 0.14812244 | No |
| RGS4 | 0.122917742 | 0.12953237 | No |
| PTGFRN | 0.119115904 | 0.12929039 | No |
| PLCD4 | 0.099402577 | 0.12350821 | No |
| OVOL2 | 0.076157562 | 0.11819029 | No |
| CD59 | 0.057318468 | 0.11280204 | No |
| PTHLH | 0.033293124 | 0.104750305 | No |
| IGSF3 | 0.027114799 | 0.10339269 | No |
| DHRS9 | 0.024083793 | 0.10236711 | No |
| TRIM6 | 0.003298137 | 0.09657376 | No |
| PELI2 | -0.036220271 | 0.0828678 | No |
| PLAU | -0.054781534 | 0.076254345 | No |
| TGFBI | -0.121444434 | 0.054034654 | No |
| CCNA1 | -0.177204713 | 0.036915954 | No |
| POLR1E | -0.187265486 | 0.035198368 | No |
| BTG3 | -0.197031721 | 0.033483073 | No |
| FAM46C | -0.208382994 | 0.031498138 | No |
| KCNG1 | -0.215771616 | 0.0309167 | No |
| SERPINE1 | -0.253528953 | 0.021678433 | No |

| S100A4 | -0.267521471 | 0.019853173 | No |
| --- | --- | --- | --- |
| LY6E | -0.289053023 | 0.015422242 | No |
| CRB3 | -0.309267402 | 0.011639564 | No |
| TMEM51 | -0.313842505 | 0.011864519 | No |
| HOXC8 | -0.315892488 | 0.013452937 | No |
| DYSF | -0.322591901 | 0.013033981 | No |
| LGALS3BP | -0.340222806 | 0.009767249 | No |
| SLC16A2 | -0.347391248 | 0.010385691 | No |
| IL1RL1 | -0.359550953 | 0.009676277 | No |
| ARHGDIB | -0.408525288 | -0.003366318 | No |
| SYT7 | -0.429696351 | -0.00518161 | No |
| IL1R2 | -0.461387247 | -0.011938759 | No |
| OTUB2 | -0.5153144 | -0.023400063 | No |
| HAS2 | -0.552036941 | -0.030115996 | No |
| GRHPR | -0.566966832 | -0.031398557 | No |
| COL6A3 | -0.594832182 | -0.03396215 | No |
| MAGEA10 | -0.619593918 | -0.036623858 | No |
| TFF2 | -0.654447377 | -0.043139428 | No |

**Supplementary Table 5.** GSEA of *HNF1B* gene signature in Bowen et al. (2009) OvCa dataset [7]. *FLRT3* is highlighted in the leading edge core set (bold); *SLC14A1* is marginally significant (truncated at 100).

| PROBE | RANK METRIC SCORE | RUNNING ES | CORE ENRICHMENT |
| --- | --- | --- | --- |
| NELL2 | 16.04439354 | 0.045874104 | Yes |
| HTRA1 | 6.155093193 | 0.058487598 | Yes |
| FZD7 | 5.499065399 | 0.07119067 | Yes |
| TSPAN8 | 5.060777664 | 0.08335961 | Yes |
| SQRDL | 4.971918106 | 0.09723976 | Yes |
| NPTX1 | 4.715147495 | 0.10875601 | Yes |
| HOXC6 | 4.487001896 | 0.11971581 | Yes |
| RGS4 | 4.328110695 | 0.13098826 | Yes |
| SLFN11 | 4.297151566 | 0.14298706 | Yes |
| SHISA3 | 4.291514874 | 0.15516149 | Yes |
| PLD5 | 4.089894295 | 0.16455448 | Yes |
| **FLRT3** | **3.907161713** | **0.17356879** | **Yes** |
| ITM2A | 3.829917431 | 0.183273 | Yes |
| CXCL6 | 3.59756875 | 0.18958063 | Yes |
| TRIM6 | 2.910607815 | 0.18323483 | Yes |
| EPB41L3 | 2.869237661 | 0.19028813 | Yes |
| SLC44A3 | 2.671762466 | 0.1916958 | Yes |
| PHACTR3 | 2.44990468 | 0.1900245 | Yes |
| AKR1C3 | 2.373610973 | 0.19355161 | Yes |
| SLITRK4 | 2.318780422 | 0.19821617 | Yes |
| FAM155A | 2.295049429 | 0.20343602 | Yes |
| TMEM27 | 2.232731581 | 0.20684794 | Yes |
| DOCK11 | 2.094020844 | 0.20578885 | Yes |
| EPDR1 | 2.027317762 | 0.20875724 | Yes |
| SLC16A2 | 1.957433939 | 0.21071094 | Yes |
| RASSF2 | 1.947236776 | 0.21584707 | Yes |
| PTHLH | 1.864156604 | 0.21662334 | Yes |
| FAM65B | 1.833304167 | 0.2208585 | Yes |
| **SLC14A1** | **1.688186169** | **0.21696137** | **No** |
| CYR61 | 1.64270854 | 0.21892595 | No |
| TMEM45A | 1.602889419 | 0.22077669 | No |
| NR2F1 | 1.422489166 | 0.21147028 | No |
| GRB14 | 1.379568219 | 0.21229902 | No |
| CA9 | 1.378596902 | 0.21614483 | No |
| SERPIND1 | 1.340410113 | 0.21681368 | No |
| GPC4 | 1.293864727 | 0.21667838 | No |
| HLA-B | 1.164816141 | 0.20836085 | No |
| ABHD10 | 1.130360961 | 0.20818946 | No |
| BASP1 | 1.030596375 | 0.20130965 | No |
| LCP1 | 1.000961781 | 0.20096003 | No |
| ARHGEF3 | 0.933593988 | 0.19663098 | No |

| ENPP4 | 0.772798598 | 0.17856447 | No |
| --- | --- | --- | --- |
| SNCA | 0.682321727 | 0.16901119 | No |
| APOBEC3C | 0.665064156 | 0.16865984 | No |
| SLC22A15 | 0.626421273 | 0.16503435 | No |
| AKR1C1 | 0.625660777 | 0.16672738 | No |
| CDH3 | 0.623450518 | 0.16817442 | No |
| PELI2 | 0.503625274 | 0.15125564 | No |
| EIF4B | 0.495122045 | 0.15147294 | No |
| PLCD4 | 0.486271441 | 0.15123352 | No |
| AGTR1 | 0.466722369 | 0.15017128 | No |
| RFTN1 | 0.39131096 | 0.13844378 | No |
| LAMB3 | 0.386880636 | 0.13897474 | No |
| FA2H | 0.366004616 | 0.13661791 | No |
| SEMA4B | 0.311142355 | 0.12892734 | No |
| CNN3 | 0.251050234 | 0.118140966 | No |
| KCNMB4 | 0.242720112 | 0.11710933 | No |
| AADAC | 0.229054958 | 0.1153196 | No |
| AKR1B10 | 0.166647568 | 0.104052246 | No |
| S100P | 0.152569383 | 0.10170829 | No |
| NNMT | 0.132835001 | 0.09830131 | No |
| GCNT3 | 0.107950538 | 0.09319341 | No |
| TFF2 | -0.011683613 | 0.068924256 | No |
| RASD1 | -0.044371516 | 0.06277177 | No |
| CCNA1 | -0.080429859 | 0.053894263 | No |
| GAGE1 | -0.130313456 | 0.04328996 | No |
| GLIPR1 | -0.136081874 | 0.042336892 | No |
| PTGFRN | -0.16459024 | 0.0369116 | No |
| C14orf37 | -0.218766615 | 0.024690775 | No |
| RRAGD | -0.237667844 | 0.020816581 | No |
| SERPINE1 | -0.27892527 | 0.01173967 | No |
| CDKN2C | -0.284475297 | 0.011498493 | No |
| PLCB1 | -0.290996999 | 0.010604885 | No |
| RELN | -0.296968639 | 0.010063891 | No |
| CD59 | -0.302829593 | 0.009491719 | No |
| FAM46C | -0.355363816 | -0.002050944 | No |
| AKR1C2 | -0.362437367 | -0.002788223 | No |
| ZIC2 | -0.383538544 | -0.007060223 | No |
| FAM20C | -0.422514051 | -0.017068736 | No |
| LDOC1 | -0.435382158 | -0.019131342 | No |
| VAMP8 | -0.457555234 | -0.023479318 | No |
| JAM3 | -0.484945387 | -0.02928287 | No |
| PLAU | -0.516204596 | -0.036195394 | No |
| IGSF3 | -0.516380012 | -0.03471896 | No |
| DHRS9 | -0.583560407 | -0.05193646 | No |
| HOXC8 | -0.590739131 | -0.05226065 | No |
| LGALS3BP | -0.64788568 | -0.06550744 | No |

| LY6E | -0.675888658 | -0.07124439 | No |
| --- | --- | --- | --- |
| CCL2 | -0.679559946 | -0.07035594 | No |
| DSP | -0.684263945 | -0.06950197 | No |
| HSPA12A | -0.696170509 | -0.070675135 | No |
| SLC20A1 | -0.733554721 | -0.077876955 | No |
| GTSF1 | -0.7356264 | -0.07630093 | No |
| FGFR4 | -0.783246398 | -0.08777061 | No |
| PCDH20 | -0.794070423 | -0.08837625 | No |
| IGDCC4 | -0.802874148 | -0.08871704 | No |
| DSE | -0.816198409 | -0.08940321 | No |
| CD70 | -0.821981072 | -0.08853896 | No |
| MX1 | -0.829278529 | -0.08822905 | No |

**Supplementary Table 6.** Consensus set of 37 core enrichment genes common between prostate and ovarian cancer GSEA ranked by order in gene list, where only the highest ranking study per gene is referenced. See also Supplementary Figure 16.

| Gene | Rank in referenced  gene list | Rank - metric score | Running ES | Study |
| --- | --- | --- | --- | --- |
| NELL2 | 1 | 16.044 | 0.046 | Bowen *et al.*, 2009 |
| CDH3 | 6 | 4.644 | 0.034 | Partheen *et al.*, 2006 |
| PLD5 | 9 | 12.000 | 0.023 | Varambally *et al.*, 2005 |
| CYR61 | 12 | 26.362 | 0.035 | Varambally *et al.*, 2005 |
| **FLRT3** | **25** | **22.500** | **0.051** | **Varambally *et al.*, 2005** |
| APOBEC3C | 34 | 11.800 | 0.018 | Taylor *et al.*, 2010 |
| SLC16A2 | 37 | 7.460 | 0.038 | Grasso *et al.*, 2012 |
| SOCS2 | 41 | 8.637 | 0.017 | Taylor *et al.*, 2010 |
| SH2D3A | 55 | 3.424 | 0.057 | Partheen *et al.*, 2006 |
| RGS4 | 73 | 7.590 | 0.137 | Varambally *et al.*, 2005 |
| HTRA1 | 105 | 6.155 | 0.058 | Bowen *et al.*, 2009 |
| TRIM6 | 118 | 12.600 | 0.050 | Grasso *et al.*, 2012 |
| FZD7 | 131 | 12.500 | 0.067 | Grasso *et al.*, 2012 |
| SNCA | 148 | 11.526 | 0.030 | Grasso *et al.*, 2012 |
| SQRDL | 189 | 10.943 | 0.061 | Grasso *et al.*, 2012 |
| TSPAN8 | 218 | 5.061 | 0.083 | Bowen *et al.*, 2009 |
| FAM155A | 236 | 5.680 | 0.216 | Varambally *et al.*, 2005 |
| NEK6 | 261 | 2.582 | 0.123 | Partheen *et al.*, 2006 |
| NPTX1 | 268 | 4.715 | 0.109 | Bowen *et al.*, 2009 |
| SLFN11 | 339 | 4.297 | 0.143 | Bowen *et al.*, 2009 |
| SLC44A3 | 385 | 9.558 | 0.128 | Grasso *et al.*, 2012 |
| EPDR1 | 413 | 3.801 | 0.199 | Tomlins *et al.*, 2007 |
| ANPEP | 430 | 5.162 | 0.117 | Taylor *et al.*, 2010 |
| ITM2A | 437 | 2.295 | 0.146 | Partheen *et al.*, 2006 |
| FA2H | 453 | 2.277 | 0.162 | Partheen *et al.*, 2006 |
| CXCL6 | 548 | 3.598 | 0.190 | Bowen *et al.*, 2009 |
| EPB41L3 | 626 | 4.606 | 0.162 | Taylor *et al.*, 2010 |
| RASSF2 | 916 | 6.726 | 0.229 | Varambally *et al.*, 2005 |
| AKR1C3 | 1009 | 6.385 | 0.259 | Varambally *et al.*, 2005 |
| PLAT | 1031 | 1.759 | 0.179 | Partheen *et al.*, 2006 |
| CA9 | 1049 | 1.747 | 0.191 | Partheen *et al.*, 2006 |
| SLITRK4 | 1304 | 2.319 | 0.198 | Bowen *et al.*, 2009 |
| TMEM27 | 1396 | 2.233 | 0.207 | Bowen *et al.*, 2009 |
| DOCK11 | 1544 | 2.094 | 0.206 | Bowen *et al.*, 2009 |
| SLC7A8 | 1694 | 1.382 | 0.216 | Partheen *et al.*, 2006 |
| TGFA | 1728 | 1.364 | 0.234 | Partheen *et al.*, 2006 |
| FAM65B | 1809 | 1.833 | 0.221 | Bowen *et al.*, 2009 |

**Supplementary Table 7.** Tested associations between top ovarian cancer risk SNP rs757210 and CpG probes surrounding HNF1B in n=231

HGS ovarian cancer samples with 450K methylation array data and Agilent gene expression data (Wang et al. 2014). Figure 3A is a graphical representation of these data.

| **Probe** | **Estimate** | **Est. Lower CI** | **Est. Upper CI** | **Std Error** | **p-value** | **Genomic co-ordinate** |  | **Gene** | **CpG Island** |
| --- | --- | --- | --- | --- | --- | --- | --- | --- | --- |
| cg11485655 | 0.0004386 | -0.181 | 0.1819 | 0.09256 | 0.9962 | 36032204 |  |  |  |
| cg14694075 | 0.01047 | -0.171 | 0.1919 | 0.09257 | 0.9101 | 36059830 | **HNF1B** |  |  |
| cg11866296 | 0.02437 | -0.1564 | 0.2052 | 0.09225 | 0.7919 | 36061601 | **HNF1B** |  |  |
| cg04136369 | -0.0699 | -0.2509 | 0.1111 | 0.09237 | 0.45 | 36070600 | **HNF1B** |  |  |
| cg21250756 | 0.09241 | -0.08699 | 0.2718 | 0.09154 | 0.3138 | 36077300 | **HNF1B** |  |  |
| cg16672659 | -0.2385 | -0.4161 | -0.06097 | 0.09058 | **0.009039** | 36098223 | **HNF1B** |  | N Shelf |
| cg15246719 | -0.08947 | -0.2684 | 0.08943 | 0.09127 | 0.328 | 36102254 | **HNF1B** |  | Island |
| cg12134754 | -0.03565 | -0.217 | 0.1457 | 0.09254 | 0.7004 | 36102394 | **HNF1B** |  | Island |
| cg24712484 | -0.1112 | -0.2911 | 0.06877 | 0.09181 | 0.2272 | 36102575 | **HNF1B** |  | Island |
| cg03433642 | -0.06439 | -0.2432 | 0.1144 | 0.0912 | 0.4809 | 36102740 | **HNF1B** |  | Island |
| cg09679923 | -0.05434 | -0.2312 | 0.1226 | 0.09026 | 0.5478 | 36103036 | **HNF1B** |  | Island |
| cg17652435 | -0.08159 | -0.2587 | 0.09552 | 0.09036 | 0.3675 | 36103066 | **HNF1B** |  | Island |
| cg03348978 | -0.04373 | -0.2222 | 0.1348 | 0.09107 | 0.6315 | 36103230 | **HNF1B** |  | Island |
| cg02435495 | -0.001038 | -0.1805 | 0.1784 | 0.09155 | 0.991 | 36103289 | **HNF1B** |  | Island |
| cg05110178 | -0.1313 | -0.3083 | 0.0457 | 0.09032 | 0.1473 | 36103953 | **HNF1B** |  | Island |
| cg11862993 | -0.09243 | -0.2695 | 0.08469 | 0.09037 | 0.3075 | 36103978 | **HNF1B** |  | Island |
| cg04917276 | -0.1633 | -0.3396 | 0.01291 | 0.08992 | 0.07062 | 36104585 | **HNF1B** |  | Island |
| cg04433035 | -0.1484 | -0.3263 | 0.0294 | 0.09073 | 0.1032 | 36105064 | **HNF1B** |  | N Shore |
| cg05222347 | -0.1119 | -0.2898 | 0.06588 | 0.09073 | 0.2185 | 36105117 | **HNF1B** |  | N Shore |
| cg02335804 | -0.1329 | -0.3121 | 0.04632 | 0.09143 | 0.1475 | 36105239 | **HNF1B** |  | N Shore |
| cg12788467 | -0.1815 | -0.3603 | -0.002777 | 0.09121 | **0.04774** | 36105335 | **HNF1B** |  | Island |
| cg13230606 | -0.184 | -0.3626 | -0.005297 | 0.09116 | **0.04475** | 36105337 | **HNF1B** |  | Island |
| cg19378036 | -0.1858 | -0.3644 | -0.007124 | 0.09114 | **0.04269** | 36105364 | **HNF1B** |  | Island |
| cg14487292 | -0.1983 | -0.3765 | -0.02016 | 0.09091 | **0.03015** | 36105517 | **HNF1B** |  | Island |
| cg04198914 | -0.1598 | -0.3387 | 0.01917 | 0.09129 | 0.08147 | 36106025 | **HNF1B** |  | S Shore |
| cg16179589 | 0.05649 | -0.1246 | 0.2376 | 0.0924 | 0.5416 | 36107292 |  |  | S Shore |

**Supplementary Table 8 A**. Associations between top ovarian cancer SNP rs757210 and expression probes in the surrounding 1 Mb region

(adjusted for age) in n=182 high-grade serous ovarian cancer cases with Agilent gene expression data and COGS genotypes (Wang et al. 2014) [9].

| **Probe** | **Estimate** | **Est. lower CI** | **Est. upper CI** | **Std error** | **p-value** | **Gene** | **Genomic coordinates** |
| --- | --- | --- | --- | --- | --- | --- | --- |
| A_24_P109962 | 0.1452 | -0.05837 | 0.3488 | 0.1039 | 0.1683 |  | chr17: 35501096-35501060 |
| **A_24_P330822** | **-0.1343** | **-0.3405** | **0.07181** | **0.1052** | **0.2032** | **HNF1B** | **chr17: 36059175-36059116** |
| A_24_P878366 | -0.1172 | -0.3235 | 0.08902 | 0.1052 | 0.2667 |  | chr17: 35846866-35846807 |
| A_23_P141434 | -0.1034 | -0.31 | 0.1031 | 0.1054 | 0.3278 | ACACA | chr17: 35445931-35444374 |
| A_23_P65157 | 0.1012 | -0.1043 | 0.3067 | 0.1048 | 0.3357 |  | chr17: 35766390-35766331 |
| A_23_P335388 | 0.08388 | -0.1194 | 0.2872 | 0.1037 | 0.4198 | C17orf78 | chr17: 35749525-35749584 |
| A_32_P215318 | 0.08526 | -0.1214 | 0.2919 | 0.1055 | 0.4199 | ACACA | chr17: 35687296-35687237 |
| A_24_P779605 | 0.07821 | -0.1282 | 0.2847 | 0.1053 | 0.4588 | SOCS7 | chr17: 36561724-36561783 |
| A_32_P111852 | 0.0747 | -0.1318 | 0.2812 | 0.1053 | 0.4791 | SOCS7 | chr17: 36561734-36561793 |
| A_23_P207537 | -0.0716 | -0.2774 | 0.1342 | 0.105 | 0.4962 | DUSP14 | chr17: 35873238-35873297 |
| A_32_P153423 | -0.06752 | -0.2737 | 0.1387 | 0.1052 | 0.5218 | DDX52 | chr17: 35970039-35969980 |
| A_24_P179033 | -0.06636 | -0.2729 | 0.1402 | 0.1054 | 0.5297 |  | chr17: 35497926-35497867 |
| A_24_P1929 | 0.05743 | -0.1494 | 0.2642 | 0.1055 | 0.869 | DDX52 | chr17: 35980949-35979857 |
| A_23_P351695 | 0.04789 | -0.1587 | 0.2545 | 0.1054 | 0.6501 | SYNRG | chr17: 35901158-35901099 |
| A_32_P9575 | -0.04296 | -0.2486 | 0.1627 | 0.1049 | 0.6827 | MRPL45 | chr17: 36454586-36455369 |
| A_23_P164210 | -0.04261 | -0.2488 | 0.1636 | 0.1052 | 0.686 | TBC1D3F | chr17: 36294855-36294914 |
| A_24_P690235 | -0.04018 | -0.2464 | 0.1661 | 0.1052 | 0.7031 | LOC440434 | chr17: 36351939-36351880 |
| A_23_P66664 | 0.03686 | -0.1697 | 0.2434 | 0.1054 | 0.7269 | TADA2A | chr17: 35822207-35825575 |
| A_23_P66658 | 0.031 | -0.175 | 0.237 | 0.1051 | 0.7684 | TADA2A | chr17: 35836941-35837000 |
| **A_23_P207557** | **0.0295** | **-0.1775** | **0.2365** | **0.1056** | **0.7083** | **HNF1B** | **chr17: 36089085-36089026** |
| A_23_P207541 | 0.01806 | -0.1887 | 0.2248 | 0.1055 | 0.8642 | SYNRG | chr17: 36089085-36089026 |
| A_23_P396626 | 0.01681 | -0.186 | 0.2196 | 0.1035 | 0.8711 | SYNRG | chr17: 35878116-35878057 |
| **A_23_P409287** | **0.01468** | **-0.199** | **0.2213** | **0.1054** | **0.8894** | **HNF1B** | **chr17: 36046809-36046750** |
| A_24_P367211 | 0.01344 | -0.1923 | 0.2192 | 0.105 | 0.8983 | SOCS7 | chr17: 36552120-36552179 |
| A_23_P118660 | -0.00855 | -0.2156 | 0.1985 | 0.1056 | 0.9356 | DDX52 | chr17: 35973928-35973869 |
| A_23_P113623 | -0.006283 | -0.2134 | 0.2008 | 0.1057 | 0.9527 | MRPL45 | chr17: 36478912-36478971 |
| A_32_P43711 | 0.002609 | -0.2033 | 0.2085 | 0.1051 | 0.9802 | SOCS7 | chr17: 36555823-36555882 |

**Supplementary Table 8 B**. Associations between top ovarian cancer SNP rs757210 and expression probes in the surrounding 1 Mb region

(adjusted for age) in n=21 clear cell serous ovarian cancer cases with Agilent gene expression data and COGS genotypes (Cicek et al. 2013) [10].

| **Probe** | **Estimate** | **Est. lower CI** | **Est. upper CI** | **Std error** | **p-value** | **Gene** | **Genomic co-ordinates** |
| --- | --- | --- | --- | --- | --- | --- | --- |
| A_ 24_P878366 | -0.8491 | -1.564 | -0.1345 | 0.3646 | 0.03171 |  | chr17: 035846866-035846807 |
| A_ 24_P690235 | -0.8375 | -1.566 | -0.1087 | 0.3719 | 0.03702 | LOC440434 | chr17: 36351939-36351880 |
| **A_ 23_P409287** | **0.6229** | **-0.01866** | **1.264** | **0.3273** | **0.07316** | **HNF1B** | **chr17: 36046809-36046750** |
| **A_ 23_P207557** | **0.5784** | **-0.04437** | **1.201** | **0.3177** | **0.08538** | **HNF1B** | **chr17: 36089085-36089026** |
| A_24_P330822 | 0.5641 | -0.09709 | 1.225 | 0.3373 | 0.1118 | HNF1B | chr17: 36059175-36059116 |
| A_23_P207537 | 0.5581 | -0.2197 | 1.336 | 0.3969 | 0.1766 | DUSP14 | chr17: 35873238-35873297 |
| A_24_P1929 | 0.4903 | -0.2439 | 1.224 | 0.3746 | 0.2070 | DDX52 | chr17: 35980949-35979857 |
| A_23_P335388 | -0.4957 | -1.263 | 0.2714 | 0.3914 | 0.2215 | C17orf78 | chr17: 35749525-35749584 |
| A_32_P153423 | 0.3599 | -0.3726 | 1.092 | 0.3738 | 0.3483 | DDX52 | chr17: 35970039-35969980 |
| A_23_P118660 | 0.3594 | -0.3903 | 1.109 | 0.3825 | 0.3599 | DDX52 | chr17: 35973928-35973869 |
| A_32_P215318 | 0.3511 | -0.4593 | 1.161 | 0.4135 | 0.4070 | ACACA | chr17: 35687296-35687237 |
| A_23_P396626 | 0.325 | -0.4314 | 1.081 | 0.3859 | 0.4108 | SYNRG | chr17: 35878116-35878057 |
| A_23_P66664 | 0.3202 | -0.4453 | 1.086 | 0.3906 | 0.4231 | TADA2A | chr17: 35822207-35825575 |
| A_23_P141434 | 0.313 | -0.4989 | 1.125 | 0.4142 | 0.4597 | ACACA | chr17: 35445931-35444374 |
| A_32_P43711 | 0.2611 | -0.4939 | 1.016 | 0.3852 | 0.5065 | SOCS7 | chr17: 36555823-36555882 |
| A_23_P113623 | 0.274 | -0.5245 | 1.073 | 0.4074 | 0.5097 | MRPL45 | chr17: 36478912-36478971 |
| A_32_P65157 | -0.2124 | -1.031 | 0.6065 | 0.4178 | 0.6173 |  | chr17: 35766390-035766331 |
| A_24_P779605 | 0.1954 | -0.6257 | 1.017 | 0.4189 | 0.6465 | SOCS7 | chr17: 36561724-36561783 |
| A_24_P179033 | 0.1599 | -0.6539 | 0.9738 | 0.4152 | 0.7046 |  | chr17: 35497926-035497867 |
| A_23_P351695 | -0.1158 | -0.8446 | 0.6131 | 0.3719 | 0.7591 | SYNRG | chr17: 35901158-35901099 |
| A_24_P367211 | 0.1147 | -0.6922 | 0.9216 | 0.4117 | 0.7837 | SOCS7 | chr17: 36552120-36552179 |
| A_24_P109962 | 0.1045 | -0.6732 | 0.8822 | 0.3968 | 0.7952 |  | chr17: 35501096-35501060 |
| A_23_P207541 | 0.09772 | -0.6488 | 0.8442 | 0.3809 | 0.8004 | SYNRG | chr17: 35875336-35875277 |
| A_32_P111852 | 0.102 | -0.7234 | 0.9273 | 0.4211 | 0.8114 | SOCS7 | chr17: 36561734-36561793 |
| A_23_P66658 | -0.05227 | -0.8482 | 0.7437 | 0.4061 | 0.8990 | TADA2A | chr17: 35836941-35837000 |
| A_32_P9575 | 0.04265 | -0.7782 | 0.8635 | 0.4188 | 0.9200 | MRPL45 | chr17: 36454586-36455369 |
| A_23_P164210 | 0.004302 | -0.7404 | 0.749 | 0.3800 | 0.9911 | TBC1D3F | chr17: 36294855-36294914 |

**References**
